# Local DNA dynamics shape mutational patterns of mononucleotide repeats in human genomes

**DOI:** 10.1093/nar/gkv364

**Published:** 2015-04-20

**Authors:** Albino Bacolla, Xiao Zhu, Hanning Chen, Katy Howells, David N. Cooper, Karen M. Vasquez

**Affiliations:** 1 Division of Pharmacology and Toxicology, College of Pharmacy, Dell Pediatric Research Institute, The University of Texas at Austin, 1400 Barbara Jordan Boulevard, Austin, TX 78723, USA; 2 Texas Advanced Computing Center, Austin, TX 78758-4497, USA; 3 Department of Chemistry, George Washington University, 725 21st Street, NW, Washington, DC 20052, USA; 4 Institute of Medical Genetics, School of Medicine, Cardiff University, Cardiff CF14 4XN, UK

## Abstract

Single base substitutions (SBSs) and insertions/deletions are critical for generating population diversity and can lead both to inherited disease and cancer. Whereas on a genome-wide scale SBSs are influenced by cellular factors, on a fine scale SBSs are influenced by the local DNA sequence-context, although the role of flanking sequence is often unclear. Herein, we used bioinformatics, molecular dynamics and hybrid quantum mechanics/molecular mechanics to analyze sequence context-dependent mutagenesis at mononucleotide repeats (A-tracts and G-tracts) in human population variation and in cancer genomes. SBSs and insertions/deletions occur predominantly at the first and last base-pairs of A-tracts, whereas they are concentrated at the second and third base-pairs in G-tracts. These positions correspond to the most flexible sites along A-tracts, and to sites where a ‘hole’, generated by the loss of an electron through oxidation, is most likely to be localized in G-tracts. For A-tracts, most SBSs occur in the direction of the base-pair flanking the tracts. We conclude that intrinsic features of local DNA structure, i.e. base-pair flexibility and charge transfer, render specific nucleotides along mononucleotide runs susceptible to base modification, which then yields mutations. Thus, local DNA dynamics contributes to phenotypic variation and disease in the human population.

## INTRODUCTION

Changes in human genomic DNA in the form of base substitutions and insertions/deletions (indels) are essential to ensure population diversity, adaptation to the environment, defense from pathogens and self-recognition; they are also a critical source of human inherited disease and cancer. On a genome-wide scale, base substitutions result from the combined action of several factors, including replication fidelity, lagging versus leading strand DNA synthesis, repair, recombination, replication timing, transcription, nucleosome occupancy, etc., both in the germline and in cancer (1–4). On a much finer scale [(over a few base pairs (bp)], rates of base substitutions may be strongly influenced by interrelationships between base–protein and base–base interactions. For example, the mutator role of activation-induced deaminase (AID) in B-cells during class-switch recombination and somatic hypermutation (5) targets preferentially cytosines within WRC (W: A|T; R: A|G) sequences (6), whereas apolipoprotein B mRNA editing enzyme, catalytic polypeptide-like (APOBEC) overexpression displays a preference for base substitutions at cytosines in TCW contexts (7). Other examples, such as the induction of C→T transitions at CG:CG dinucleotides by cytosine-5-methylation and the role of UV light in promoting base substitutions at pyrimidine dimers have been well documented (reviewed in (4,8)). More recently, complex patterns of base substitution at guanosines in cancer genomes have been found to correlate with changes in guanosine ionization potentials as a result of electronic interactions with flanking bases (9), suggesting a role for electron transfer and oxidation reactions in sequence-dependent mutagenesis. However, despite these advances, the increasing number of sequence-dependent patterns of mutation noted in genome-wide sequencing studies has met with a lack of understanding of most of the underlying mechanisms (10). Thus, a picture is emerging in which mutations are often heavily dependent on sequence-context, but for which our comprehension is limited.

Mononucleotide repeats comprise blocks of identical base pairs (A|T or C|G; hereafter referred to as A-tracts and G-tracts) and display distinct features: they are abundant in vertebrate genomes; mutations within the tracts occur more frequently than the genome-wide average; mutations generally increase with increasing tract length; length instability is a hallmark of mismatch repair-deficiency in cancers; and sequence polymorphism within the general population has been linked to phenotypic diversity (11–15). Thus, mononucleotide repeats appear ideal for addressing the question of sequence-dependent mutagenesis since base pairs within the tracts are flanked by identical neighbors. Both historic and recent investigations concur with the conclusion that a major source of mononucleotide repeat polymorphism is the occurrence of slippage (i.e. repeat misalignment) during semiconservative DNA replication, which gives rise to the addition or deletion of repeat units (11,12). An additional and equally important source of mutation has recently been suggested to arise from errors in DNA replication by translesion synthesis DNA polymerases, such as pol η and pol κ (13), also on slipped intermediates, leading to single base substitutions.

A key question that remains unanswered in these studies and which is relevant to the issue of sequence context-dependent mutagenesis is whether all base pairs within mononucleotide repeats display identical susceptibility to single base changes and whether indels (which are consequent to DNA breakage) occur randomly within the tracts.

Herein, we combine bioinformatics analyses on mononucleotide repeat variants from the 1000 Genomes Project and cancer genomes with molecular dynamics simulations and hybrid quantum mechanics/molecular mechanics calculations to address the question of sequence-dependent mutagenesis within these tracts. We show that mutations along both A-tracts and G-tracts are highly non-uniform. Specifically, both base substitutions and indels occur preferentially at the first and last bp of A-tracts, whereas they are concentrated between the second and third G:C base pairs in G-tracts. These positions coincide with the most flexible base pairs for A-tracts and with the preferential localization of a ‘hole’ that results when one electron is lost due to an oxidation reaction anywhere along G-tracts. Thus, despite the uniformity of sequence composition, mutations occur in a sequence-dependent context at homopolymeric runs according to a hierarchy that is imposed by both local DNA structural features and long-range base–base interactions. We also show that the repair processes leading to base substitution must differ between A- and G-tracts, since in the former, but not in the latter, base substitutions occur predominantly in the direction of the base immediately flanking the tracts. Additional sequence-dependent patterns of mutation are likely to arise from studies of more heterogeneous sequence combinations, possibly involving other aspects intrinsic to the structure of DNA.

## MATERIALS AND METHODS

### Datasets

A- and G-tracts were retrieved from the hg19 human genome assembly (or from exons) available at UCSC (http://hgdownload.soe.ucsc.edu) using custom scripts. The 1KGP SNV and indel data were obtained from version 3 of the integrated variant call set at ftp://ftp.1000genomes.ebi.ac.uk/vol1/ftp/release/20110521. For the Neanderthal genomes, ∼18.5M contigs sequenced from bone of three Neanderthal individuals were downloaded from ftp://hgdownload.cse.ucsc.edu/gbdb/hg18/neandertal/seqAlis/. Approximately half of the numbers of tracts present in the hg19 assembly were found in the three Neanderthal samples. For gene enrichment analyses, genes with A- and G-tracts were retrieved from http://hgdownload.cse.ucsc.edu/goldenpath/hg19/database/ and the resulting NM_* entries were used as input to DAVID (http://david.abcc.ncifcrf.gov) for gene enrichment analyses. The charts generated were used to obtain two types of information: (i) the terms with a Benjamini-corrected *P*-value <E-10 for A-tracts and <E-05 for G-tracts; and (ii) the terms with a Benjamini-corrected *P*-value <0.05 that were in common between the A- and G-tracts. Nucleotides from A- and G-tracts intersecting transcription factor-bound chromatin immunoprecipitated genomic segments were obtained from http://hgdownload.cse.ucsc.edu/goldenpath/hg19/encodeDCC/wgEncodeRegTfbsClustered/. For A- and G-tracts along well-positioned nucleosomes, file GSE36979_mnase_mids_combined_147.wig.gz deposited in GEO with accession number GSE36979 (16) was downloaded from http://www.ncbi.nlm.nih.gov/geo/query/acc.cgi?acc=GSE36979. We mapped all tract coordinates matching those covered by the GSE36979_mnase_mids_combined_147 file intervals. Cancer SBSs were downloaded from ftp://data.dcc.icgc.org/, ftp://ftp.sanger.ac.uk/pub/cancer/AlexandrovEtAl and https://tcga-data.nci.nih.gov/tcga/findArchives.htm, and sorted to exclude duplicate SBSs. HGMD data were obtained from HGMD^®^ Professional release 2014.2 (http://www.biobase-international.com/product/hgmd). SBSs (*n* = 3805), 1-bp deletions (*n* = 1540) and 1-bp insertions (*n* = 1324) occurring in A- and G-tracts of 4–13 bp were extracted using the ‘Advanced’ data analysis tool.

### MD simulations

MD simulations were performed as described (17). Briefly, we describe the interactions between DNA molecules and the environment (e.g. water and counter ions) by the AMBER parm99 force field with parmbsc0 nucleotide modifications (parmbsc0). We solvated the DNA fragments (G[6] and G[9] capped by CGC and A[6] and A[9] capped by CGCGCG at both ends to mitigate end effects) in a cubic water box using the SOLVATE module implemented in Visual Molecular Dynamics (VMD) (18). Each side of the box extended 10 Å away from any solute atom and water molecules within a distance of 2.6 Å of any solute atom were deleted. Sodium ions were randomly added to the water box in each case to neutralize the net charge. Periodic boundary conditions were used and the periodic box was filled with water molecules based on the modified TIP3P potential. MD simulations were carried out with the molecular dynamics package NAMD (19) at 298K. A 2-ns MD simulation at 298K with an isothermal-isobaric (NPT) ensemble was performed to equilibrate the system. During the equilibration, the position of DNA atoms was kept fixed with harmonic constraints, which were released stepwise in the final 1 ns. A second equilibration was then carried out with a NPT ensemble for 4 ns at 298K without constraints, followed by 100 ns production runs for G-tracts and 32 ns for A-tracts. Langevin dynamics was employed with a damping coefficient *γ* = 5 ps^−1^ (20). Pressure was maintained at 1 atm by the Langevin piston method, with a piston period of 200 fs, a damping time constant of 100 fs and a piston temperature of 298K (20). Non-bonded interactions were calculated with a group-based cutoff and a switching function at each time step; full electrostatics was employed using the particle-mesh Ewald method, with 1 Å grid width at every other step. Covalent bonds involving hydrogen atoms were held rigid with the SHAKE algorithm, allowing a 2 fs time step.

### DFT simulations

Vertical ionization potentials of double-stranded G[6] and G[9] DNA fragments were calculated by comparing the energies before and after removing an electron from the static solvated structures. To account for structural fluctuation and thus to ensure statistical reliability, a total of 40 solvated DNA structures for both G[6] and G[9] were randomly selected from the 100-ns MD trajectories. A hybrid quantum mechanics/molecular mechanics (QM/MM) approach was employed (21) with the Gaussian electrostatic coupling scheme (22) to attain a balance between accuracy and efficiency. Specifically, the DNA molecules were modeled by the density functional theory (DFT) (23), whereas water molecules and sodium counterions were described by the modified TIP3P and AMBER parm99 potentials, respectively. All DFT simulations were performed using the CP2K software (24) with the Goedecker-Teter-Hutter (GTH) pseudopotential (25), the Perdew-Burke-Ernzehof (PBE) exchange-correlation functional (26) and the polarized-valence-double-ζ (PVDZ) basis set (27). Charge redistribution upon vertical ionization was examined by tracking the differences on the density-derived atomic partial charges (DDAPC) (28), grouped by DNA base pairs. The calculated charge difference is always negative since the charge per bp after electron loss is subtracted from that of the non-perturbed state.

## RESULTS

### Mononucleotide repeat variation is defined by tract length and flanking base composition

**Figure 1.**
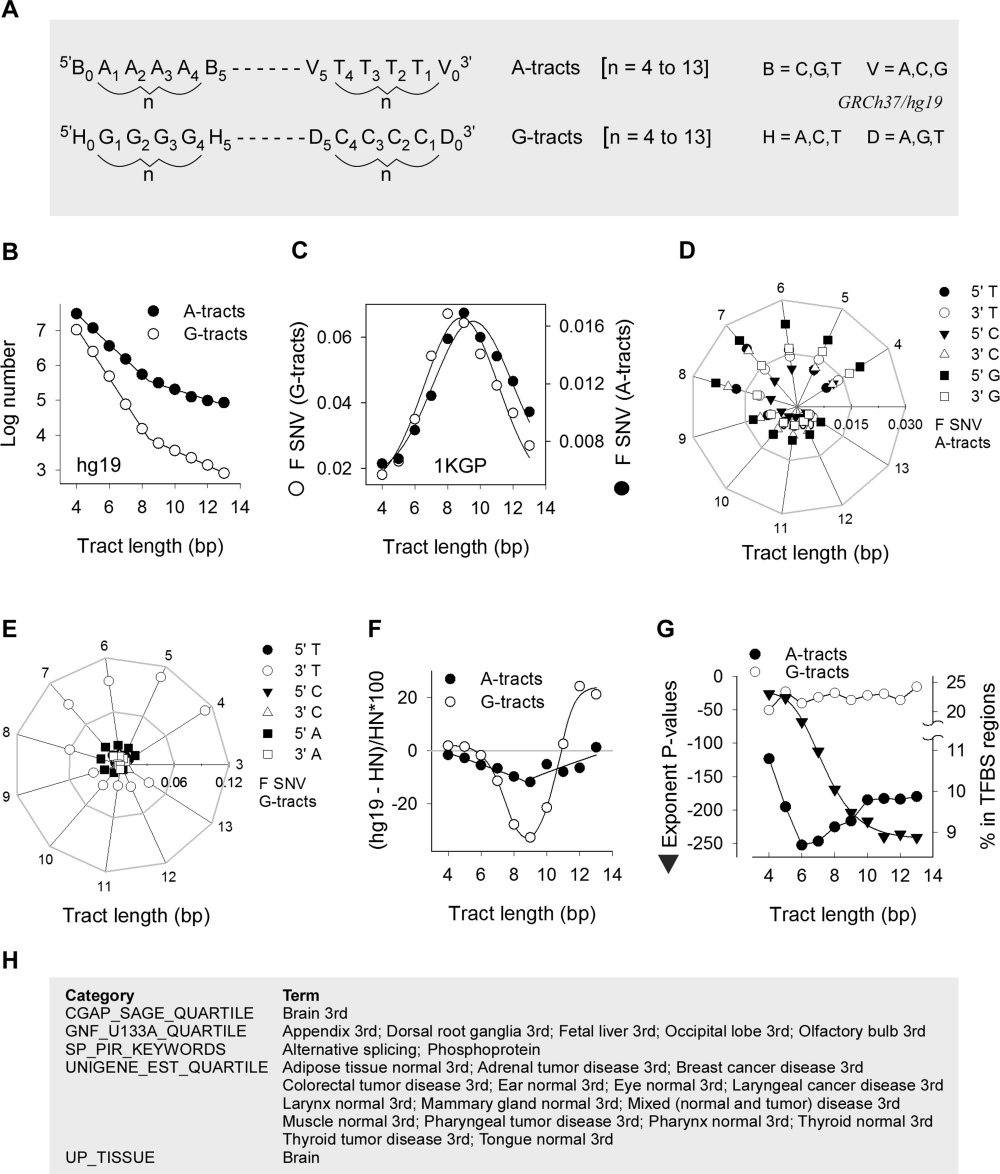
Mononucleotide repeat variation, evolutionary conservation and association with transcription. (**A**) The search algorithm was designed to retrieve runs of As or Ts (A-tracts) and Gs or Cs (G-tracts) length *n* (*n* = 4 to 13), along with their 5′ (*n* = 0) and 3′ (*n* = *n* + 1) nearest neighbors from hg19. Tract bases were numbered 5′ to 3′ with respect to the purine-rich sequence. The panel exemplifies the nomenclature for A- and G-tracts of length 4. (**B**) Logarithmic plot of the number of A-tracts (closed circles) and G-tracts (open circles) in hg19 as a function of length. (**C**) Normalized fractions of polymorphic tracts (F SNV) (number of SNVs divided by both hg19 number of tracts and n) from the 1KGP for A-tracts (closed circles) and G-tracts (open circles). (**D**) Radial plot of SNVs in the 1KGP at the 5′ and 3′ nearest neighbors of A-tracts. Periphery, tract length; horizontal axis, scale for the fraction of SNVs (F SNV). (**E**) Radial plot of SNVs in the 1KGP at the 5′ and 3′ nearest neighbors of G-tracts. (**F**) Percent difference in the numbers of A-tracts (closed circles) and G-tracts (open circles) between syntenic regions of hg19 and HN genomes. (**G**) The exponents of Benjamini-corrected *P*-values for A-tract-containing genes enriched in transcription-factor binding sites plotted as a function of A-tract length (triangles); each value represents the median of the top 11 USCS_TFBS terms. The percent A-tracts (closed circles) and G-tracts (open circles) intersecting genomic regions pulled-down by chromatin immunoprecipitation using antibodies against transcription factors are plotted as a function of tract length. (**H**) List of gene enrichment terms with a Benjamini-corrected *P*-value of <0.05 in common between genes containing A- and G-tracts of lengths 4–13, excluding the UCSC_TFBS terms.

We define mononucleotide repeats in the GRCh37/hg19 (hg19) human genome assembly as uninterrupted runs of A:T and G:C base pairs (hereafter referred to as A-tracts and G-tracts, respectively) from 4 to 13 base pairs in length (Figure 1A). We retrieved a total of 48,767,945 A-tracts and 13,633,781 G-tracts, both of which displayed a biphasic distribution with an inflection point between tract lengths of 8 and 9 (bp) and with the number of runs declining with length more dramatically for G-tracts than for A-tracts (Figure 1B), as noted previously (29). Both the number of short tracts and the extent of decline varied with flanking base composition, TA[n]T runs being two- to three-fold more abundant than CA[n]Cs (Supplementary Figure S1A) and AG[n]As declining the most rapidly (Supplementary Figure S1B). Thus, mononucleotide runs exist as a collection of separate pools of sequences in extant human genomes, each maintained at distinctive rates of sequence stability, as determined by factors such as bp composition (A:T versus G:C), tract length and flanking sequence composition.

We examined the extent of sequence variation in the human population by mapping 38,878,546 single nucleotide variants (SNVs) from 1092 haplotype-resolved genomes (the 1000 Genomes Project, 1KGP) (30) to the hg19 A- and G-tracts. The normalized fractions of polymorphic tracts (F SNV) were greater for G-tracts than A-tracts and both displayed Gaussian-type distributions, with maxima of 0.067 for G-tracts of length 8 and 0.017 for A-tracts of length 9 (Figure 1C). CA[n]C and AG[n]A runs displayed the highest F SNV values for A- and G-tracts, respectively (Supplementary Figure S1C and D), with F SNV values for AG[n]As attaining ∼0.10 at length 8. We conclude that flanking base composition influences the rates of SNV within mononucleotide runs and, as a consequence, their representation in the reference human genome.

F SNV values at the flanking 5′ and 3′ bp were similar between A- and G-tracts, except for minor differences for the least represented (i.e. longest) tracts and did not exceed 0.02 (Supplementary Figure S1E). These fractions are expected to be greater than at more distant positions from the tracts, based on previous data (29). SNVs at G-tracts, but not at A-tracts, were more frequent than at flanking base pairs. F SNVs for base pairs flanking short (≤8 bp) tracts were at least twice as high as those flanking long tracts; F SNVs also displayed distinct sequence preference with most (∼0.1) variants occurring at Ts 3′ of G-tracts (Figure 1D and E). In summary, SNVs at mononucleotide runs do not increase monotonically with length but peak at 8–9 bp. This behavior mirrors the genomic distributions, both with respect to the total number of tracts (Figure 1B) and the subsets flanked by specific-sequence combinations (Supplementary Figure S1A–D). Variation at flanking base pairs also displayed a biphasic pattern centered at a length of 8–9 bp, with a greater chance of variation adjacent to G- than A-tracts and with characteristic sequence preferences.

### Long tracts are evolutionarily conserved and associated with high transcription

To assess whether more variable monosatellite runs (Figure 1C) might have undergone a greater reduction in number in extant humans relative to extinct hominids, we compared the number of A- and G-tracts between syntenic regions of five individuals comprising hg19 and three Neanderthal (HN) specimens (31). The difference between hg19 and HN was very small (<±2%) for the short tracts, but it displayed more negative values in hg19 with increasing tract length, which reached a maximum of −11.8 and −32.7% for A- and G-tracts, respectively, of length 9. Beyond this threshold, the numbers of tracts converged for A-tracts, whereas they were more abundant in hg19 for G-tracts >11 bp (Figure 1F). In summary, the largest difference in the number of mononucleotide runs between hg19 and HN sequences was centered at 9 bp for both A- and G-tracts, suggesting that the length distributions (Figure 1A and Supplementary Figure S1A and B) reflect distinct rates of evolutionary gains and losses due to differential sequence mutability (Figure 1C) as a function of length and flanking sequence composition (12).

The fact that long (>9 bp) mononucleotide runs display low variability in the human population (Figure 1C) and sequence conservation during evolutionary divergence (Figure 1F) raises the possibility that they might serve functional roles. Through gene enrichment analyses, we found that genes containing A- and G-tracts were enriched for genes associated with the term ‘UCSC_TFBS’, which pertains to transcripts harboring frequent transcription factor binding sites (32,33). For A-tract-containing genes, the median *P*-values for the top 11 UCSC_TFBS terms decreased from 2.95E-26 for tracts of length 4 to 5.22E-241 for tracts of length 13 (Figure 1G). The percent of A-tracts intersecting genomic fragments amplified from chromatin immunoprecipitation using transcription-factor binding antibodies (32,33) also increased from 8.7 to 9.9 from length 6 to 13, whereas it was constant (mean ± SD, 22.4 ± 1.1) for G-tracts (Figure 1G). For gene classes excluding ‘UCSC_TFBS’, a search for categories enriched at *P* < 0.05 and common to all A- and G-tract-containing genes returned a set of 25 terms, 22 of which were associated with high levels of tissue-specific gene expression (Figure 1H). In summary, these analyses extend prior work (14) supporting a role for mononucleotide tracts in enhancing gene expression, a function that for A-tracts appears to increase with increasing tract length.

### Repeat variability is highly skewed

**Figure 2.**
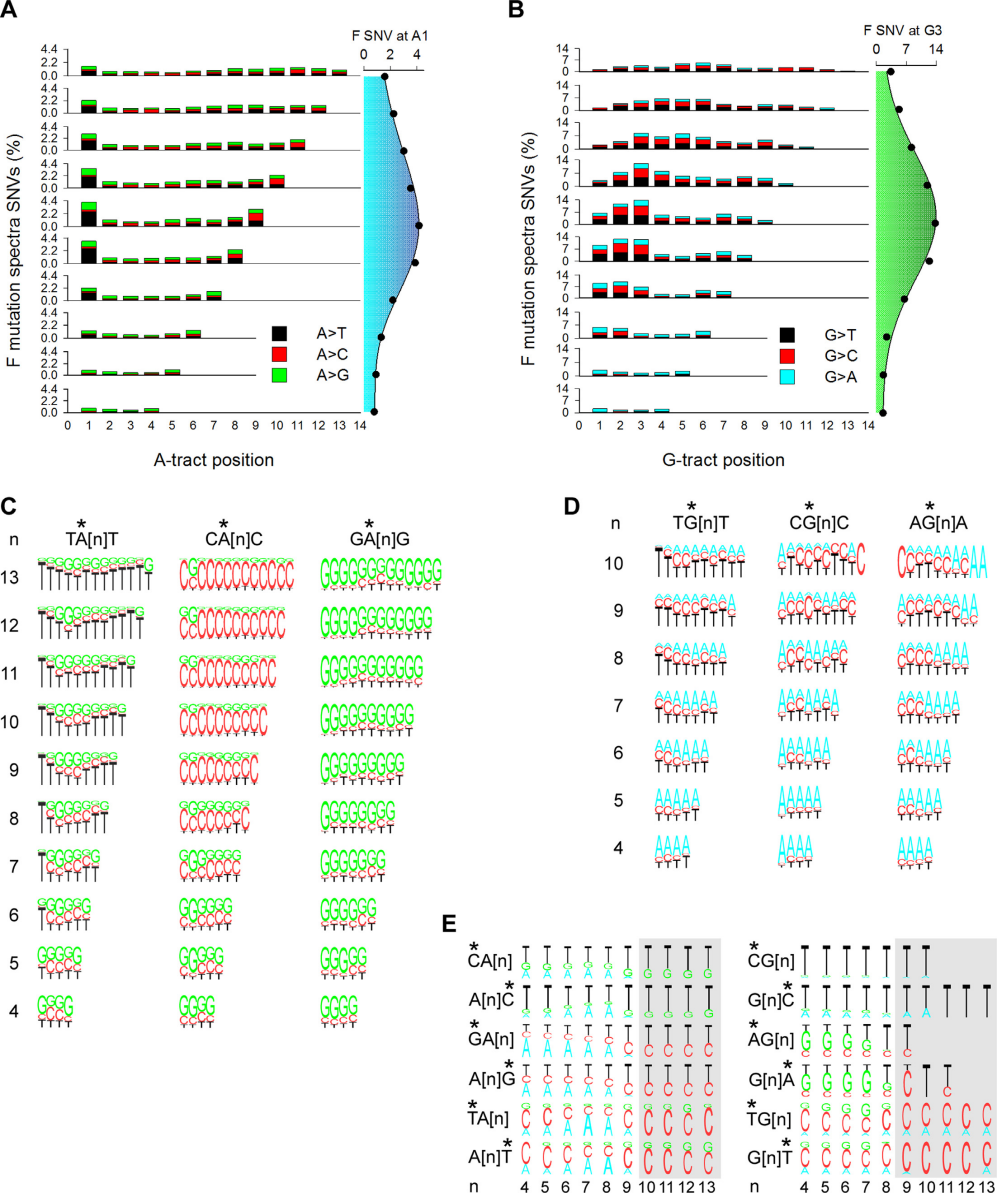
Population variation spectra. (**A**) Variation spectra of A-tracts. Percent (number of SNVs at each position divided by the number of tracts in hg19 × 100) of A→T (black), A→C (red) and A→G (green) SNVs in the 1KGP dataset (left). Percent SNVs at A_1_ as a function of tract length (right). (**B**) Variation spectra of G-tracts. As in panel A with G→T (black), G→C (red) and G→A (cyan) (left). Percent SNVs at G_3_ as a function of tract length (right). (**C**) Percent A→T, A→C and A→G transitions at each position along A-tracts (stars) preceded and followed by a T (TA[n]T, left), C (CA[n]C), center) and G (GA[n]G, right) as a function of tract length. (**D**) Percent G→T, G→C and G→A transitions at each position along G-tracts (stars) preceded and followed by a T (TG[n]T, left), C (CG[n]C), center) and A (AG[n]A, right) as a function of tract length. (**E**) Percent transitions at base pairs (stars) preceding or following A-tracts (left) and G-tracts (right) as a function of tract length (*n*). *, mutated position.

Next we addressed whether bp along A- and G-tracts display equal probability and type of variation. In the 1KGP dataset, the number of SNVs at each position along both A- and G-tracts of length 4 was within a two-fold difference (144,000–240,000); for both types of sequence, transitions (i.e. A→G and G→A) were the predominant (51–78%) type of base substitution (Supplementary Figure S2A and B). However, with increasing length, the number of SNVs decreased up to 30-fold more drastically for G-tracts than for A-tracts, with increasing numbers of transversions (A→T and G→C|T) being predominant. Normalizing the data for the number of tracts genome-wide revealed that the extent of SNV varied by up to 10-fold, depending upon tract length and bp position. Specifically, the highest degree of variation was observed at the first and last A within the A-tracts (i.e. A_1_ and A_n_), which underwent up to 61% A→T and 43% A→C transversions, respectively, at length 9 (Figure 2A). Likewise, for G-tracts, the most polymorphic sites were G_3_, followed by G_2_, for mid-size tracts of 8–10 bp, with 44% G→C transversions at G_3_ for tracts of length 8 (Figure 2B). Thus, the extent of SNV at mononucleotide runs is grossly skewed in human genomes, both along the sequence itself and across tract length, which must account for the bell-shape behavior in F SNV for the tracts as a whole (Figure 1C).

We assessed whether SNV hypervariability was associated with specific combinations of nearest neighbors. For A-tracts flanked 5′ by a T, C or G, the highest percentage of SNVs was observed at A_1_ when preceded by a T, which reached 7.9% for TA[n] tracts of length 9 (Supplementary Figure S2C). By contrast, for 3′ T, C or G, the greatest effect was elicited by a C, with the highest percentage (7.1%) of SNVs at A_n_ for A[n]C tracts of length 9 (Supplementary Figure S2D). Therefore, flanking base pairs play a critical role both in the spectra and frequencies of SNVs at A-tracts. More detailed plots along A-tracts either preceded (Supplementary Figure S2E), followed (Supplementary Figure S2F) or preceded and followed (Figure 2C) by a T, C or G revealed the dramatic and long-range (up to 9–10 bp for the longest tracts, higher than the value of 4 bp predicted by mathematical models of slippage (11)) influence of flanking base pairs on variation spectra, in which up to 95% of the changes were in the direction of the base flanking the tract. Because the number of A-tracts preceded or followed by a specific base varies by up to three-fold (Supplementary Figure S2G), we conclude that for A-tracts, the overall mutation fractions and spectra are the result of at least three variables; length, position along the tract, and base composition of the 5′ and 3′ nearest-neighbors.

For G-tracts flanked 5′ by a T, C or A, high percentages (10–12%) of SNVs were observed at G_1_ for tracts preceded by a C, an effect that decreased with increasing tract length (Supplementary Figure S3A). This result, together with an exceedingly low number of G→A transitions at G_1_ for tracts not preceded by a C (Supplementary Figure S3C) relative to all tracts (Supplementary Figure S2B), is consistent with the known high mutability of CG:CG dinucleotides as a result of cytosine-5 methylation (9). The hypermutability at G_2_ was observed preferentially for tracts preceded by an A, and to a lesser extent T, whereas that at G_3_ was insensitive to flanking sequence composition. Likewise, G-tracts flanked 3′ by a T, C or A did not display marked sequence-dependent effects (Supplementary Figure S3B). Detailed plots of the SNV spectra along G-tracts either preceded (Supplementary Figure S3D), followed (Supplementary Figure S3E), or preceded and followed (Figure 2D) by a T, C or A revealed a noticeable effect only for 5′ T in association with G→T substitutions at G_1_ for tracts of length ≥8. Thus, despite a consistent over-representation of G-tracts flanked 5′ by a T (Supplementary Figures S3F and S1B), which must account for the high absolute number of SNVs at G_1_ for TG[n] relative to AG[n] and CG[n] (Supplementary Figure S3G), nearest-neighbor base composition seems to play a lesser role in SNV spectra at G-tracts than at A-tracts.

With respect to SNVs at the flanking 5′ and 3′ nearest positions, no B→A or H→G substitutions (Figure 1A) were found above a length threshold of 9 for A-tracts and 8 for G-tracts (Figure 2E, gray shading) out of 5969 SNVs, implying that tract expansion by recruiting flanking base pairs is disfavored at these lengths. In summary, base substitution along mononucleotide repeats is strongly skewed towards the edges of A-tracts and within the 5′ half of G-tracts, with frequencies that peak at midsize lengths (8–9 bp). For A-tracts ≥7 bp, base substitution occurred almost exclusively in the direction of the flanking nearest-neighbors. Finally, base substitution at flanking bases did not contribute to tract expansion for mononucleotide runs longer than 8–9 bp.

### Insertions and deletions display length and positional preference

**Figure 3.**
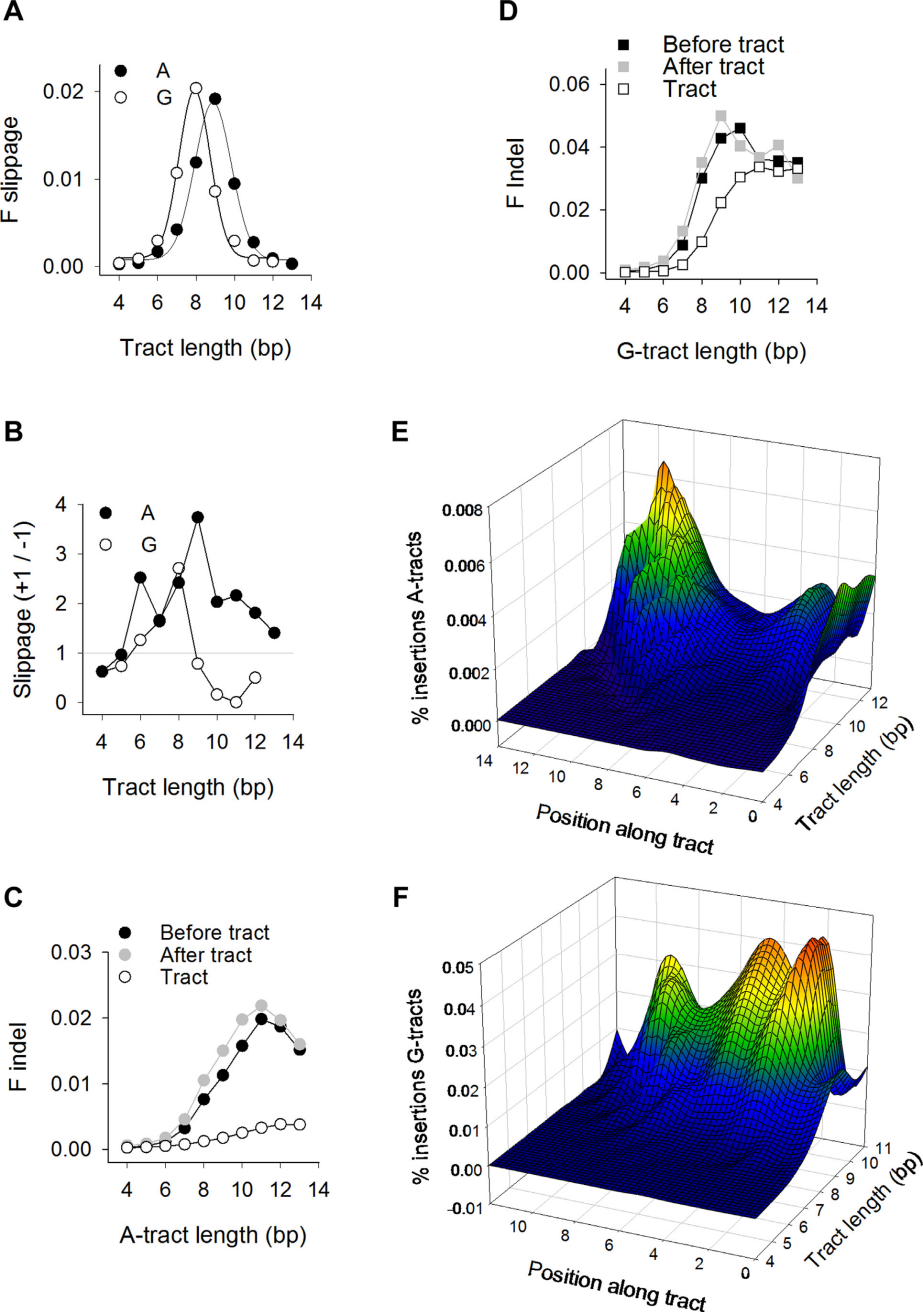
Population insertions and deletions. (**A**) Normalized fractions of A-tracts (closed circles) and G-tracts (open circles) displaying +/−1 bp slippage in the 1KGP dataset as a function of tract length. Data were obtained by dividing the number of events by both the number of hg19 tracts and tract length (*n*). (**B**) Ratio of the number of +1 to −1 slippage for A-tracts (closed circles) and G-tracts (open circles). (**C**) Indels at A-tracts. For positions along the tracts (‘Tract’), ‘F Indel’ is the ratio between the number of indels and the number of tracts in hg19 multiplied by tract length. For the positions immediately flanking the tracts genomic coordinates (‘Before tract’ and ‘After tract’), ‘F Indel’ is the ratio between the number of indels and the number of tracts in hg19. (**D**) Indels at G-tracts, calculated as described in panel C. (**E**) Heatmap representation of insertions along A-tracts. The percent insertions (i.e. the number of insertions at each position divided by the number of tracts in hg19) (y-axis) plotted as a function of location (x-axis) from position 0 (insertion between the bp 5′ to the tract and the first bp of the tract) to position *n* + 1 (insertion between the bp 3′ to the last bp of the tract and the following bp) (see Figure 1A) and as a function of tract length (z-axis). (**F**) Heatmap representation of insertions along G-tracts.

In addition to SNVs, mononucleotide runs are polymorphic in length as a result of indels. Herein, we consider separately two types of indels: one in which tract length changes by ±1 and flanking bp composition is not altered (slippage); the other comprising all other cases involving the addition or removal of 1–200 bp (indels). Slippage is a widely accepted mutational mechanism (11–12,34), whereby DNA replication errors at reiterated DNA motifs cause changes in the number of motifs (most often +/−1). The normalized fractions of slippage in the 1KGP dataset peaked at lengths of 8 bp for A-tracts and 9 bp for G-tracts (Figure 3A), generating bell-shaped curves similar to those observed for SNVs (Figure 1C) and with no differences in the highest fraction of ‘slipped’ tracts, which peaked at ∼0.02. By contrast, +1 slippage occurred more frequently than −1 slippage at A-tracts (Figure 3B). These results support recent studies on microsatellite repeats (12) and contrast with previous conclusions that slippage increases monotonically with tract length, and that the extent of slippage differs between A- and G-tracts (35,36).

With respect to indels, the normalized fractions were low (<1 × 10^−3^) along short (4–6 bp) A- and G-tracts, but rose to a plateau for longer tracts as reported earlier (11); this plateau was 10-fold higher for G-tracts (∼0.03) than for A-tracts (∼0.003) (Figure 3C and D). Indels also occurred more frequently (up to six-fold for A-tracts of length 11) at nearest-neighboring base pairs (‘Before tract’ and ‘After tract’ in Figure 3C and D) than along the tracts. Thus, contrary to SNVs and slippage, indels increased to a plateau with mononucleotide tract length.

We analyzed in detail the locations of insertions along the tracts and the flanking positions with respect to the 5′ to 3′ orientation of the tracts (Figure 1A). The normalized fractions demonstrated that insertions peaked at the 3′, and to a lesser extent 5′, ends of the longest A-tracts (Figure 3E), but remained low. For G-tracts, insertions occurred most efficiently at two locations (G_2–3_ and G_5_) (Figure 3F), they increased with tract length (up to ∼0.04), and attained ∼10-fold higher values than for A-tracts. In conclusion, insertion sites at A- and G-tracts followed the patterns observed for SNVs (Figure 2A and B), suggesting that factors associated with local DNA dynamics sensitize specific bases along the tracts to genetic alteration, inducing both SBS and indels.

### Base pair flexibility and charge localization map to sites of sequence changes

**Figure 4.**
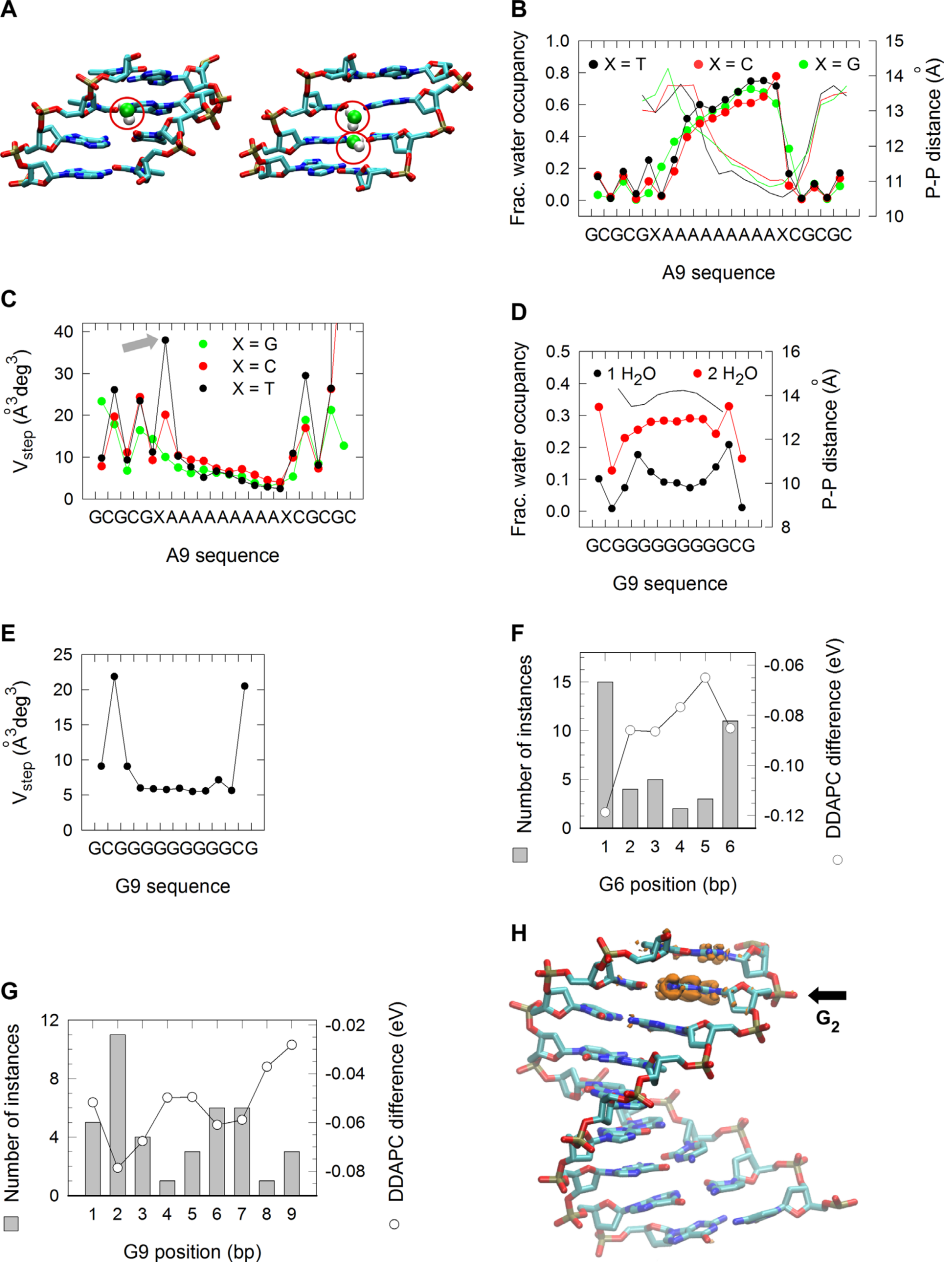
MD and QM/MM simulations. (**A**) Molecular modeling of one (left) and two (right) minor groove water bridge coordination. (**B**) Fraction of one-water bridge occupancy (left axis) at A[9] DNA sequences flanked 5′ and 3′ by a T (black circles), C (red circles) or G (green circles). Minor groove widths (right axis), as determined from intrastrand phosphate-to-phosphate distances. (**C**) V_step_ for A[9] DNA sequences, determined as the product of the square root of the eigenvalues (λ_i_) described by the six bp step parameters shift, slide, rise, tilt, roll and twist; i.e. $V_{step} = \prod\nolimits_{i = 1}^6 {\sqrt {\lambda _i } }$. (**D**) Fraction of one- (black circles) and two-water (red circles) bridge occupancy (left axis) at G[9] DNA sequences. Minor groove widths (right axis), as assessed from intrastrand phosphate-to-phosphate distances. (**E**) V_step_ for G9 DNA sequences. (**F**) Average charge redistribution (open circles and right axis) for G[6] DNA structures upon vertical ionization, examined by calculating the difference on the density-derived atomic partial charges (DDAPC) for the neutral and negatively charged states. Histogram of the number of instances (left axis) in which the largest charge redistribution occurred at a specific position along the G[6] structures. (**G**) DDAPC for G[9] DNA structures (open circles and right axis) and histogram of the number of instances (left axis) in which the largest charge redistribution occurred at a specific position. (**H**) VMD rendering of a G[9] DNA structure displaying hole localization at G_2_. Capped base pairs were removed for clarity.

To elucidate elements of intrinsic DNA dynamics that may be responsible for the biases in SNV and insertion sites, we performed molecular dynamics (MD) and hybrid quantum mechanics/molecular mechanics (QM/MM) simulations on model A[6], A[9], G[6] and G[9] duplex DNA fragments. We focused on water bridge coordination (Figure 4A), bp step flexibility, and for the G[6] and G[9], charge localization, as these properties are known to impact the susceptibility of DNA to base damage, repair and mutation. The fractions of one water coordination increased along the A[9] and A[6] structures in a 5′ to 3′ direction, irrespective of flanking sequence composition, in concert with a decrease in minor groove width (Figure 4B and Supplementary Figure S4A) as predicted (37). V_step_, a measure of bp structural fluctuation, displayed a prominent peak of ∼40 Å^3^deg^3^ at the 5′-TA-3′ step for both structures (Figure 4C and Supplementary Figure S4B), which together with low water occupancy points to 5′-TA-3′ being a preferred location for base modification and mutation. In the G[9] and G[6] structures water coordination involved mostly two-water bridges due to wide (∼14 Å) minor grooves (Figure 4D and Supplementary Figure S4C), whereas flexibility was modest (∼20–22 Å^3^deg^3^, Figure 4E and Supplementary Figure S4D). Thus, bp dynamics are likely to impact mutations at A-tracts to a greater extent than at G-tracts. Guanine has the lowest ionization potential (IP) of all four bases and IP further decreases at guanine runs, rendering them targets for electron loss, charge localization, oxidation and eventually mutation (4,38). Because after electron loss the ensuing charge (hole) can migrate along the DNA double-helix and relocalize at specific guanines, we addressed whether the preferred sites of mutation along G-tracts, i.e. G_2–3_ and G_5_, would also be preferred sites for charge localization. The QM/MM determinations indicated that whereas for the short G[6] fragment the difference in the density-derived atomic partial charges (DDAPC) (i.e. the hole) localized most often (∼50%) to the first position (Figure 4F), for the long G[9] fragment charge localization shifted downstream (mostly to the second, but also to positions 6–7, Figure 4G). Importantly, the charge was found exclusively around the guanine rings (Figure 4H). Thus, the two main sites of sequence change along G-tracts, i.e. G_2–3_ and G_5_, coincide with positions where charge localization and hence one-electron oxidation reactions is predicted to occur most frequently. In summary, bp flexibility at A-tracts and charge transfer at G-tracts likely represent intrinsic DNA features underlying the bias in SNV and insertions at mononucleotide runs in human genomes.

### Position and orientation along nucleosome core particles modulate sequence variation

**Figure 5.**
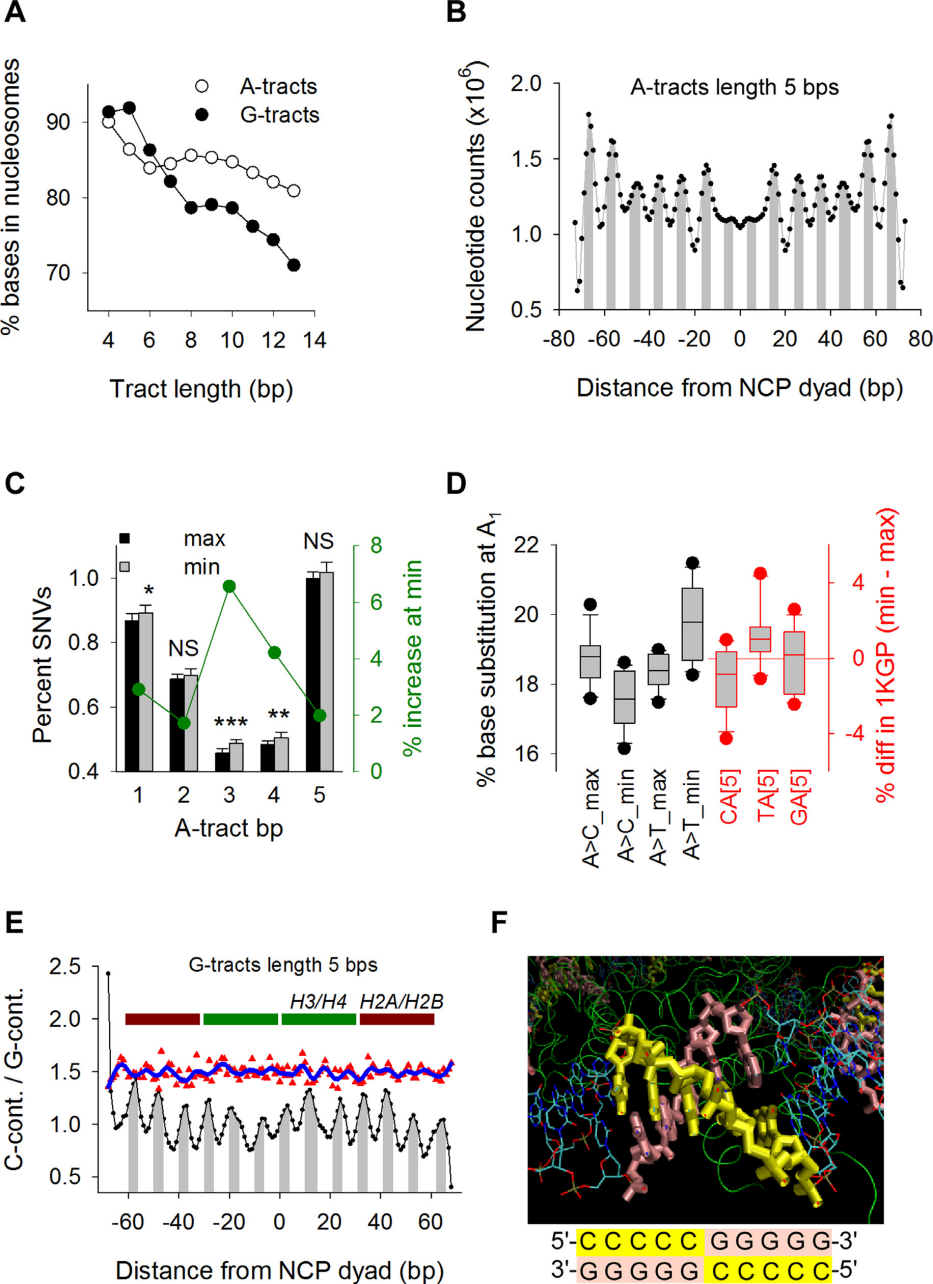
Positioning along nucleosome core particles. (**A**) Percent of A-tract (open circles) and G-tract (closed circles) base pairs in hg19 overlapping with well-positioned NCP genomic coordinates as a function of tract length. (**B**) Counts of base pairs in hg19 A-tracts of length 5 overlapping with NCPs genomic regions as a function of distance from the histone octamer dyad axis. Minor groove-inward-facing regions (gray) were derived from the X-ray crystal structure of NCP147 (41). (**C**) Percent SNVs in the 1KGP dataset (left axis) at every bp along A-tracts of length 5 for tracts centered at maxima (black) and minima (gray) along NCPs (Figure 5B). Percent increase (right axis) of SNVs at minima relative to maxima (green). *P*-values for paired *t*-tests: 0.013 (*), 0.002 (**) and 4.7 × 10^−6^ (***). (**D**) Whisker plots of%SNVs (left axis) at A_1_ for A-tracts of length 5 centered at maxima and minima (black) along NCPs (Figure 5B). Percent difference (right axis) in the number of A-tracts of length 5 in hg19 preceded by C, T or G (red) between those centered at minima and those centered at maxima (Figure 5B). (**E**) C-containing/G-containing ratios (see text) for G-tracts of length 5 in hg19 as a function of distance from the NCP dyad axis (black) and location of core histones (maroon and green). Peaks correspond to negative iSAT (i.e. tilt parameters multiplied by the corresponding sin θ) values (gray) (39). Ratios of%SNV at G_1_ (upshifted by 0.5 for clarity) between C-containing (5′-CCCCCG-3′ sequences on the hg19 forward strand) and G-containing (5′-CGGGGG-3′ sequences on the hg19 forward strand) (Figure 1A) CG[5] tracts mapping NCP Chip-seq genomic intervals (red) fitted by a non-parametric local regression (loess; sampling proportion, 0.100; polynomial degree, 3). (**F**) VMD rendering (top) of TATTT residues 34–38 (yellow) and the complementary AAATA residues 672–753 (pink) from the 1EQZ pdb nucleosomal crystal structure, corresponding to peak area from −40 to −36 in Figure 5E. The switch in G-tract (lengths of 5 and 7) orientation along NCPs (bottom) serves to position the C-containing strand on the outside (yellow) and, correspondingly, the G-containing strand on the inside (pink).

DNA wrapped around histones in nucleosomes is subject to local deformation (39), which may impact mutation. Thus, we analyzed the 1KGP SNVs at A- and G-tracts predicted to overlap with well-positioned nucleosome core particles (NCPs) (16). In hg19, the percentage of tracts that overlap with NCPs decreased moderately from ∼90% at length of 4 to 81% and 71% for A- and G-tracts of length 13, respectively (Figure 5A), suggesting that mononucleotide runs are not depleted in NCPs in human genomes as previously proposed (40). A-tracts of lengths 4–8 base pairs displayed distinctive peaks along the NCP surface in phase with the helical repeat of DNA (10.5 bp) and with minor grooves facing toward the inner protein core (lengths 4–5) (16) (Figure 5B and Supplementary Figure S5A). A-tracts of length of 9–13 bp exhibited only half (six) the peaks evident for the shorter tracts. For the G-tracts, only small peaks with no clear minor groove-inward-facing regions were detected (Supplementary Figure S5B).

To assess if tract-positioning along NCPs influences SNVs, we selected A-tracts of lengths 5, 7 and 9 bp and G-tracts of lengths 5 and 7 bp whose central positions coincided with either the maxima or minima (41) (Figure 5B and Supplementary Figure S5A and B) and conducted pair-wise *t*-tests (330 total) between permutations of ‘categories’, including ‘tracts centered at maxima versus minima’, ‘position along the tracts’, ‘flanking sequence composition’, ‘specific NCP locations’ and ‘tract orientation’. For A-tracts, 79/207 (38%) significant pairs were found, 68 (86%) of which were related to differences between tracts centered at maxima versus minima, with a preponderance (63%) of tests displaying increased %SNVs at minima (Supplementary Figure S5C and E). For example, %SNVs at length 5 bp were greater at minima than at maxima at each position along the A-tracts (Figure 5C). A→C substitutions at A_1_ were more abundant at maxima than at minima (mean ± SD, 18.7 ± 0.7% at max and 17.6 ± 0.8% at min; *P*-value 0.001), whereas A→T substitutions at the same position displayed the opposite trend (mean ± SD, 18.4 ± 0.5% at max and 19.8 ± 1.1% at min; *P*-value 0.0005) (Figure 5D). A-tracts of length 7 also exhibited a similar pattern at A_7_ (Supplementary Figure S5H). The percentages of CA[5] and A[7]C tracts in hg19 centered at maxima were greater than at minima and the reverse was observed for the TA[5] and A[7T] tracts (Figure 5D and Supplementary Figure S5H). Thus, we conclude that positioning along the NCP surface of both the double-helical grooves and junctions with flanking base pairs influence SNVs along A-tracts. However, this influence is complex and for the most part, difficult to predict.

For G-tracts, most pairwise comparisons (18/34, 53%) indicated SNV variation according to sequence orientation (Supplementary Figure S5F and G). In hg19, the ratio of the numbers of G-tracts of lengths 5 and 7 for which the C-containing strand coincided with the forward sequence (downstream example sequence in Figure 1A) to the numbers of G-tracts for which the G-containing strand coincided with the forward sequence (upstream example sequence in Figure 1A) (C-containing/G-containing ratios) displayed a prominent 10.5-bp oscillation in phase with iSAT (Figure 5E), a measure of ‘inside’ and ‘outside’ bases, according to the bp step tilt parameter (39). Analysis of the helical path of a 146-bp DNA fragment wrapped around histones showed that the oscillation in the C-containing/G-containing ratios corresponds to a preference for guanine bases to face the protein core (Figure 5F). We analyzed the subset of G-tracts preceded by a 5′ C (i.e. CG[5]) to assess whether SNVs at G_1_, the position known to be mutable due to CpG methylation also oscillated with the C-containing/G-containing ratios. Oscillation in SNV-C-containing/SNV-G-containing values was evident, with peaks aligning to the hg19 troughs (Figure 5E) implying that the cytosines facing the protein surface harbor more variants than those facing away. We conclude that A- and G-tracts display preferential positioning (the former) and orientation (the latter) along NCPs, which in turn modulate the rate of sequence variation.

### Mutations associated with human disease

**Figure 6.**
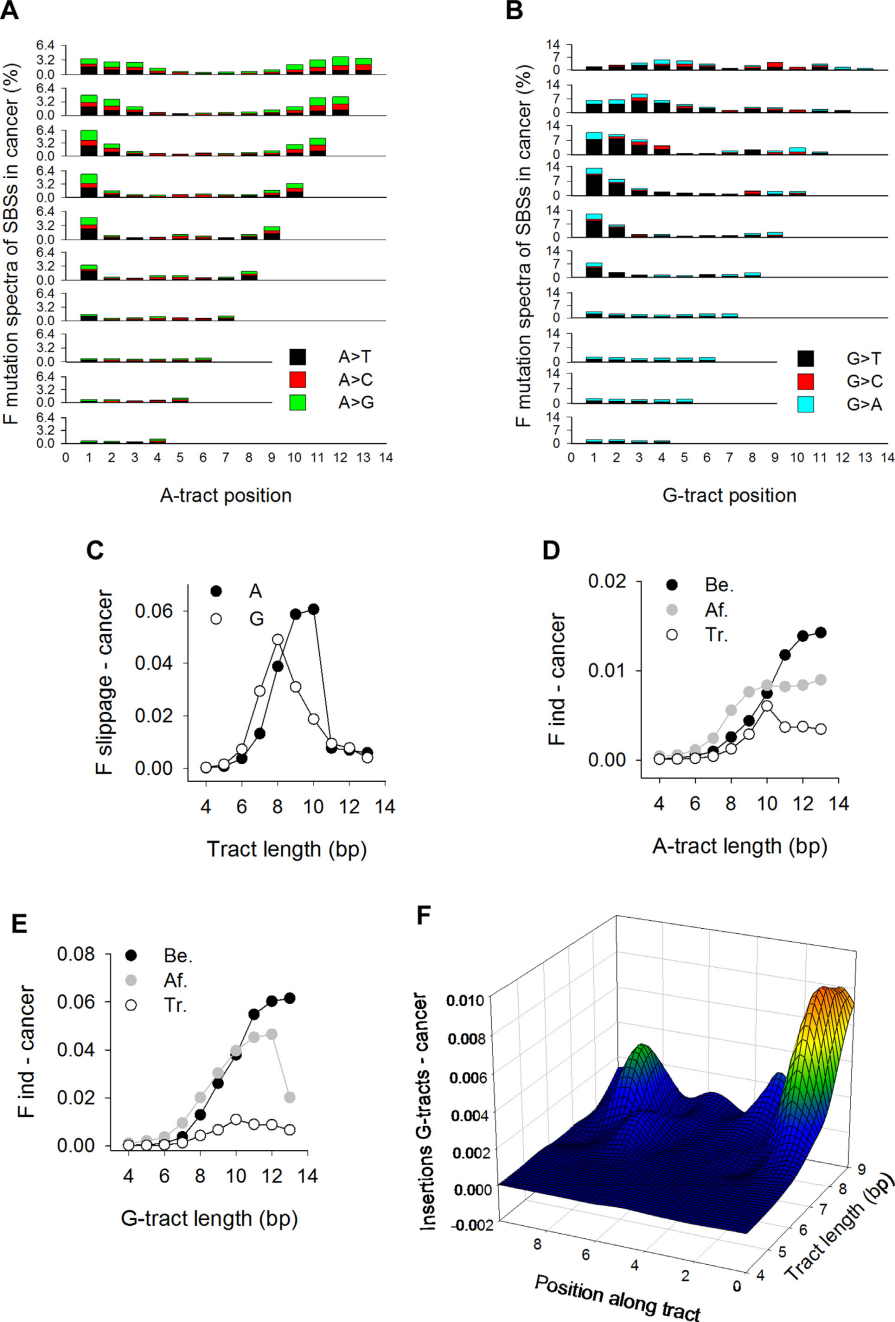
Mutation patterns in cancer genomes. (**A**) Mutation spectra for SBSs at A-tracts. Percent values were obtained by dividing the total number of SBSs at each position by the number of tracts in hg19 and then multiplying by 3.2516 to equalize the percentage of A-tracts of length 4 between the cancer genomes and the 1KGP datasets. (**B**) Mutation spectra for SBSs at G-tracts in cancer genomes. Percent values were obtained as in (A) using a multiplication factor of 3.7419. (**C**) Normalized fractions of A-tracts (closed circles) and G-tracts (open circles) displaying +/−1 bp slippage, obtained by dividing the number of events by both the number of tracts in hg19 and tract length. (**D**) Indels at A-tracts, calculated as described in Figure 3C. (**E**) Indels at G-tracts, calculated as described in Figure 3C. (**F**) Heatmap representation of insertions along G-tracts, as described in Figure 3E.

Knowing that the first and last As of long A-tracts and G_2–3_ in G-tracts are the major sites of SNV in the human population, we addressed whether these features are also discernible in mutated mononucleotide tracts associated with human genetic disease. We collected 9,450,456 unique SBSs (both SBSs and SNVs refer to single base changes) from sequenced cancer genomes and normalized the percent mutations along A- and G-tracts to enable a direct comparison with the 1KGP dataset. For A-tracts (Figure 6A and Supplementary Figure S6A), SBSs displayed the same trend as the 1KGP data (Figure 2A) with respect to the bell-shape increase in mutations at A_1_ and A_n_ and the mutation spectra, although the susceptibility to mutation as a function of tract length attained greater values (6.36% for length 11 in cancer versus 4.15% for length 9 in the 1KGP datasets at A_1_). The first and last 3 bp also harbored more SBSs than in the 1KGP dataset for tracts >7 bp, a feature that we found to be due exclusively to a large cancer dataset (42) containing high-level microsatellite instability (MSI) samples (Supplementary Figure S6B and C), which are known to result from mismatch-repair deficiency (15). Thus, A-tracts display similar patterns of base substitution between the germline and somatic cancer tissues. For G-tracts, mutation spectra were characterized by G→T transversions at tract lengths >7, particularly at G_1_, the most frequently mutated position for tracts lengths up to 11 bp (Figure 6B and Supplementary Figure S6D). This trend persisted even when the high rates of methylation-mediated deamination mutations at the CG dinucleotide were removed (Supplementary Figure S6E). Thus, mutation patterns in cancer genomes contrast with those observed in the germline, both with respect to the most mutable position (G_1_ versus G_2–3_) and the types of base substitution (G→T in cancer genomes versus G→T and G→C in the germline).

With respect to slippage, the fractions for A-tracts elicited an excess at lengths 9 and 10 bp relative to the 1KGP dataset, which was also due to the MSI-containing dataset. For G-tracts, the fractions peaked at length 8, as for the 1KGP dataset (Figures 3A and 6C), implying that the propensity to undergo slippage is indistinguishable between the germline and soma. Indels were also more abundant at flanking base pairs than along the tracts (Figure 6D and E), particularly for G-tracts of length >7, similar to the 1KGP dataset (Figure 3C and D). Detailed analyses of insertions revealed that both G_1_ and the preceding position were the most significant sites of mutation (*F*-values up to 0.08 at G_1_ for tracts of length 8) (Figure 6F). Thus, the 5′ end of long G-tracts is the most susceptible site for both SBSs and insertions in cancer genomes, in contrast to the germline where these occur within the runs, typically at G_2–3_.

**Figure 7.**
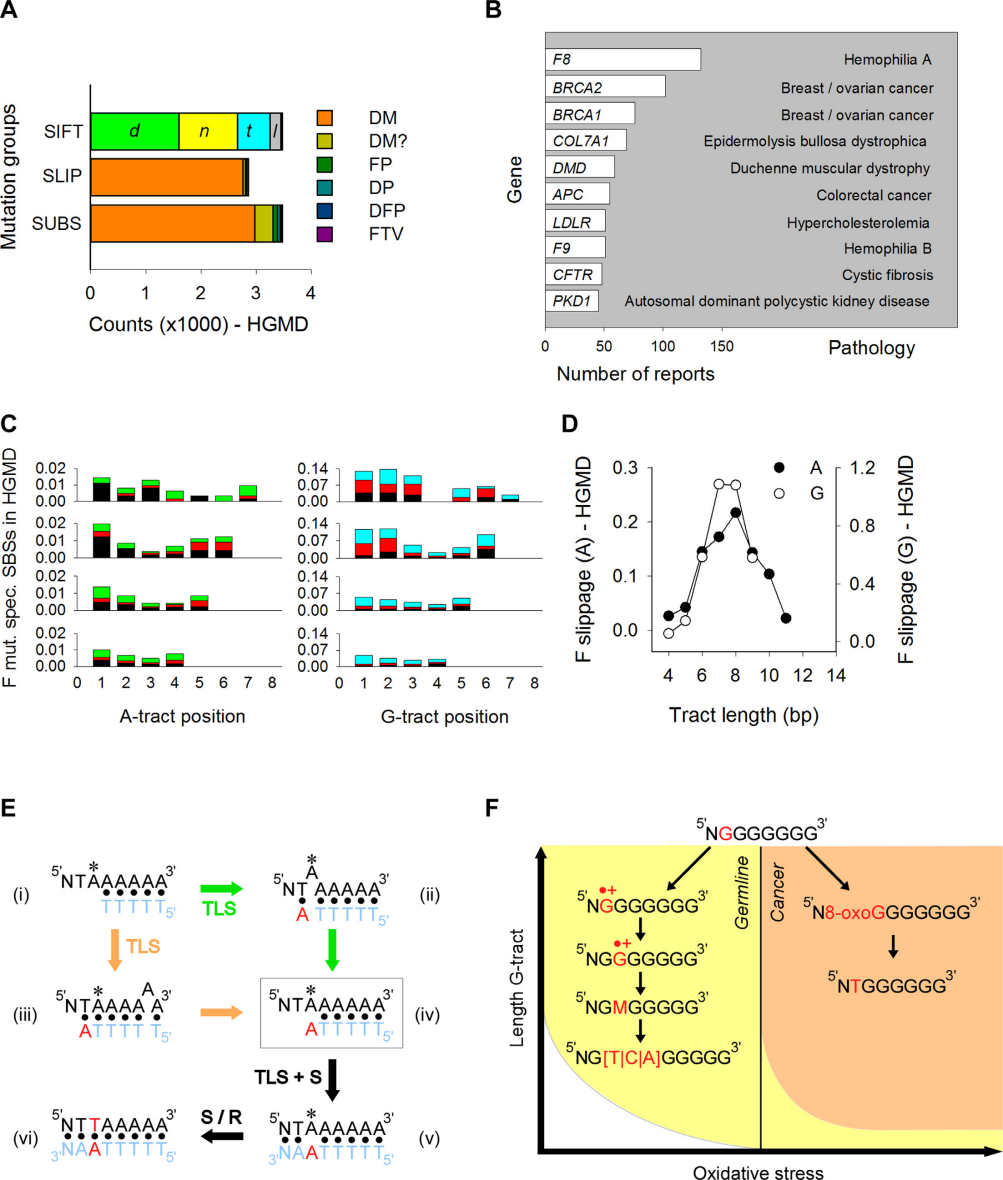
Mutation patterns in HGMD and model for sequence context-dependent changes. (**A**) Number of germline SBSs and slippage events (Slip.) at A- and G-tracts in HGMD. Gene alterations were classified as disease-causing mutation (*DM*), likely disease-causing mutation (*DM?*), disease-associated and putatively functional polymorphism (*DFP*), disease-associated polymorphism with additional supporting functional evidence (*DP*) and *invitro*/laboratory or *invivo* functional polymorphism (*FP*). Codon changes (SIFT predictor) were classified as damaging (*d*), null (*n*), tolerated (*t*) and low-confidence prediction (*l*). (**B**) The 10 most commonly reported genes in HGMD with mutations at A- and G-tracts. Various mutated tracts were generally reported for the same gene in different reports. (**C**) Mutation spectra for SBSs at A- (left) and G-tracts (right) in HGMD. Percent values were obtained by dividing the total number of SBSs at each position by the number of tracts in hg19 exons. A|G→T (black), A|G→C (red), A→G (green), G→A (cyan). (**D**) Normalized fractions of A-tracts (closed circles) and G-tracts (open circles) displaying +/−1 bp slippage, obtained by dividing the total number of events by the number of tracts in hg19 exons and by tract length. (**E**) Model for sequence context-dependent changes at A-tracts (left) and G-tracts (right). *, site of base modification.

We also extracted the mutated A- and G-tracts from the Human Gene Mutation Database (HGMD), a collection of >150,000 germline gene mutations associated with human inherited disease. A total of 1519 genes were mutated at A- or G-tracts out of a total of 3972 (38%); 3480 SBSs and 2866 slippage events were noted within these tracts, 85 and 46% of which were predicted to be disease-causing, respectively (Figure 7A and Supplementary Table S1). Ranking genes by the number of literature reports indicated that among the top 10 entries three were associated with cancer (*BRCA1, BRCA2* and *APC*), two with hemophilia (*F8* and *F9*), four with debilitating lesions of the skin (*COL71A*), muscle (*DMD*), lung (*CFTR*) and kidney (*PKD1*), with one causing hypercholesterolemia (*LDLR*) (Figure 7B). Thus, mutations within A- and G-tracts carry a high social burden by contributing to some of the most common human pathological conditions.

For both A- and G-tracts, SBSs occurred mostly at tract lengths of 4–7, with patterns more similar to those in the 1KGP than in the cancer datasets, both with respect to the location of the most mutable positions (first and last As and first/second Gs) and the types of base substitution (A→T and G→H) (Figure 7C and Supplementary Figure S6F). Likewise, slippage events peaked at tract lengths of 7–9 as observed in the 1KGP dataset (Figure 7D). In summary, the patterns of both SBSs and slippage in the HGMD dataset followed the trend observed in the 1KGP dataset, suggesting that germline variants at mononucleotide repeats leading to either population variation or human inherited disease may have arisen through similar mechanisms.

## DISCUSSION

Why are specific A:T and G:C base pairs within A- and G-tracts more susceptible to sequence changes than their identical neighbors? For A-tracts, bp flexibility may play a role. Chemical damage to DNA, such as by hydroxyl radicals has been shown to be proportional to the geometrical solvent-accessible surface of the atomic groups, which increases with DNA flexibility (43). Along A-tracts flexibility is restricted, but it is high at both the 5′ and 3′ junctions. Thus, the fact that the highest rates of mutation coincide with the highest degree of flexibility at the 5′-TA-3′ bp step is consistent with the view that this position may be susceptible to DNA damage as a result of flexibility. Other sources of DNA dynamics are also likely to be relevant, such as sugar flexibility at the junctions, which increases with tract length (44). Chemical modification at these junctions may then lead to base substitution and indels, the latter as a result of strand breaks.

With respect to SNV mutation spectra, these were found mostly in the direction of flanking base composition above a length of 7–8 bp. We interpret this behavior in terms of DNA slippage along A-tracts when attempts are made during translesion synthesis (TLS) to bypass a damaged site (Figure 7Ei). Two scenarios may be considered to account for A→T transitions at A_1_. In the first, the last tract-template base would loop out into the polymerase active site permitting base-pairing and strand elongation (Figure 7Eii) using the tract-flanking base as a template (34,45–46). In the second (Figure 7Eiii), slippage would occur behind the polymerase, prompting extension past the newly created A*:T mispair generated by primer/template misalignment. Either pathway would yield a common intermediate (Figure 7Eiv) that contains the base complementary to the junction across from the damaged site upon slippage resolution (34). Following DNA synthesis (S) and/or repair (R) (Figure 7Ev and vi), this mispair will generate a base change that is always identical to the tract-flanking base.

For G-tracts, the high rates of G→T transversions at G_1_ in cancer genomes are also consistent with preferred chemical attack at this site due to high flexibility (Figure 7F top). Direct chemical attack at a guanine is known to result in stable products, such as 8-oxo-G and Fapy-G, both of which are known to yield G→T transversions (47–50). Thus, G_1_ may be the most susceptible site for such reactions for G-tracts of lengths ≥7 (Figure 7F right), which in cancer genomes would become a mutation hotspot. In the germline, SNVs peaked inside G-tract base pairs, while mutational spectra were insensitive to flanking base composition; these events are inconsistent with a role for template misalignment and slippage as noted for A-tracts. Rather, the correspondence between hotspot mutations at G_2–3_ and G_5_ and the QM/MM simulations suggest a role for charge transfer. A large body of work during the past 20 years using computational, theoretical chemistry and biophysical techniques on short oligonucleotides, has shown that guanine is the most easily oxidizable base in DNA and that indeed a guanine radical cation can be generated through long-range hole transfer from an oxidant via one-electron oxidation mechanisms (51–55). GGG triplets were found to act as the most effective traps in hole transfer by both experimental and theoretical work (56–59), demonstrating that the resulting guanine radical cation (or its neutral deprotonated form) became rather delocalized, but it preferentially centered at the first and second G. These well-established patterns of chemical reactivity are consistent with our experimental observation of high mutation frequencies at G_1_ for short G-tracts and the results from QM/MM simulations on G6. For longer tracts, the downstream shift in mutation hotspots, i.e., G_2–3_ and G_5_, also correlate well with the charge localization predicted from QM/MM simulations, which explicitly included solvent effects and structural fluctuations. Thus, in conjunction with the constrained density functional theory (60), both the neutral and oxidized forms of a guanine nucleobase can be reliably constructed to infer the accurate determination of mutational patterns of mononucleotide repeats in human genomic DNA.

The compact organization of the sperm genome (61), and presumably low levels of oxidative stress in the germline, may enable guanine oxidization through one-electron oxidation reactions rather than by direct chemical attack, thereby favoring the formation of radical cations. A charge injected at G_1_ by electron loss would then migrate to neighboring guanines and localize at sites of low IP, such as G_2_ (Figure 7F left). Guanine radical cations are known to readily undergo further chemical modification leading to products such as 8-oxo-G, oxazolone, imidazolone, guanidinohydantoin, and spiroiminodyhydantoin (62) (M in Figure 7F), to yield G→T, G→C and G→A substitutions (4,63). Our model is in line with recent observations in which mutations at guanines within short G-runs (1–4 bp) correlate with sequence-dependent IPs at the target guanine in cancer genomes (9). Interestingly, these correlations were not observed in the germline (9). We interpret these composite observations as follows. The IP values for G-runs have been shown to decrease asymptotically with tract length, although the absolute values vary according to the methods and assumptions used (we obtained a value of 5.43 eV for both G[6] and G[9]) (64,65). We suggest that short G-runs with high IPs undergo one-electron oxidation reactions in the oxidative environment of cancer cells but would be refractory to such a mechanism in the germline (Figure 7F right yellow and left white sectors). As length increases and IP values fall, G-runs would be attacked directly by oxidants abundant in tumor cells (Figure 7F orange sector), whereas oxidation will be limited to electron loss in the germline environment (Figure 7F left yellow sector).

These models (template misalignment for A-tracts and charge transfer for G-tracts) suggest a more complex scenario for mechanisms underlying mononucleotide repeat polymorphism in the human population than recently proposed (13), in which nucleotide misincorporation by error-prone polymerases is proposed as a primary source of mutations at both A- and G-tracts. As already stated, the directionality of SNVs toward tract-flanking bases in A-tracts and the hotspot mutations at G_2–3_, supports multiple and distinct mechanisms of base substitution at mononucleotide repeats.

Our analyses highlight additional information, including the lack of mutations in the direction of tract-base composition for base pairs flanking long tracts, the association with gene expression and the preference of guanines for the inner NCP surface, and extend prior observations (12) such as the bell-shape character of base substitution and slippage, whose mechanisms remain to be fully clarified. Finally, we document the contribution of mononucleotide mutagenesis to key aspects of human pathology beyond the well-established MSI instability in cancer (15), including hemophilia and tissue degeneration. Our collective work supports the conclusion that as the human genome undergoes evolutionary diversification and along the way suffers disease-associated mutations, oxidation reactions including charge transfer may play a prominent role.

## SUPPLEMENTARY DATA

Supplementary Data are available at NAR Online.

SUPPLEMENTARY DATA
