# Supplementary material for: Local DNA dynamics shape mutational patterns of mononucleotide repeats in human genomes
Source: Nucleic Acids Res. 2015 Apr 20;43(10):5065–80. doi: 10.1093/nar/gkv364 (PMC4446427; doi:10.1093/nar/gkv364)
Supplement: SUPPLEMENTARY DATA [file supp_43_10_5065__index.html]

Local DNA dynamics shape mutational patterns of mononucleotide repeats in human genomes — Local DNA dynamics shape mutational patterns of mononucleotide repeats in human genomes — SUPPLEMENTARY DATA 

# Local DNA dynamics shape mutational patterns of mononucleotide repeats in human genomes

## SUPPLEMENTARY DATA

**Files in this Data Supplement:**

- SUPPLEMENTARY DATA
- SUPPLEMENTARY DATA
