# Supplementary material for: Local DNA dynamics shape mutational patterns of mononucleotide repeats in human genomes
Source: Nucleic Acids Res. 2015 Apr 20;43(10):5065–80. doi: 10.1093/nar/gkv364 (PMC4446427; doi:10.1093/nar/gkv364)
Supplement: SUPPLEMENTARY DATA [file supp_gkv364_nar-00635-f-2015-File009.docx]

**Supplementary Figure S1**


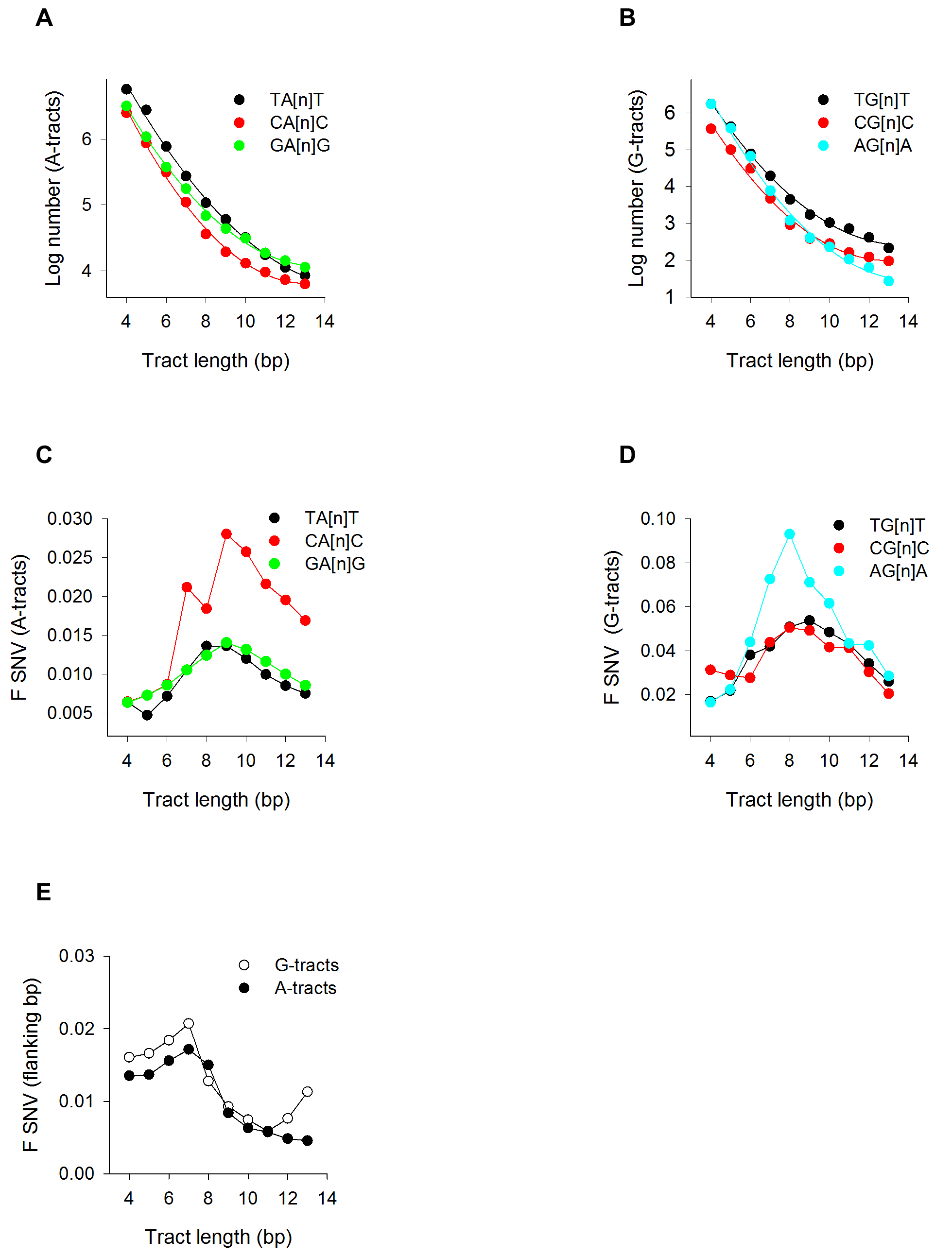


**Supplementary Figure S2**


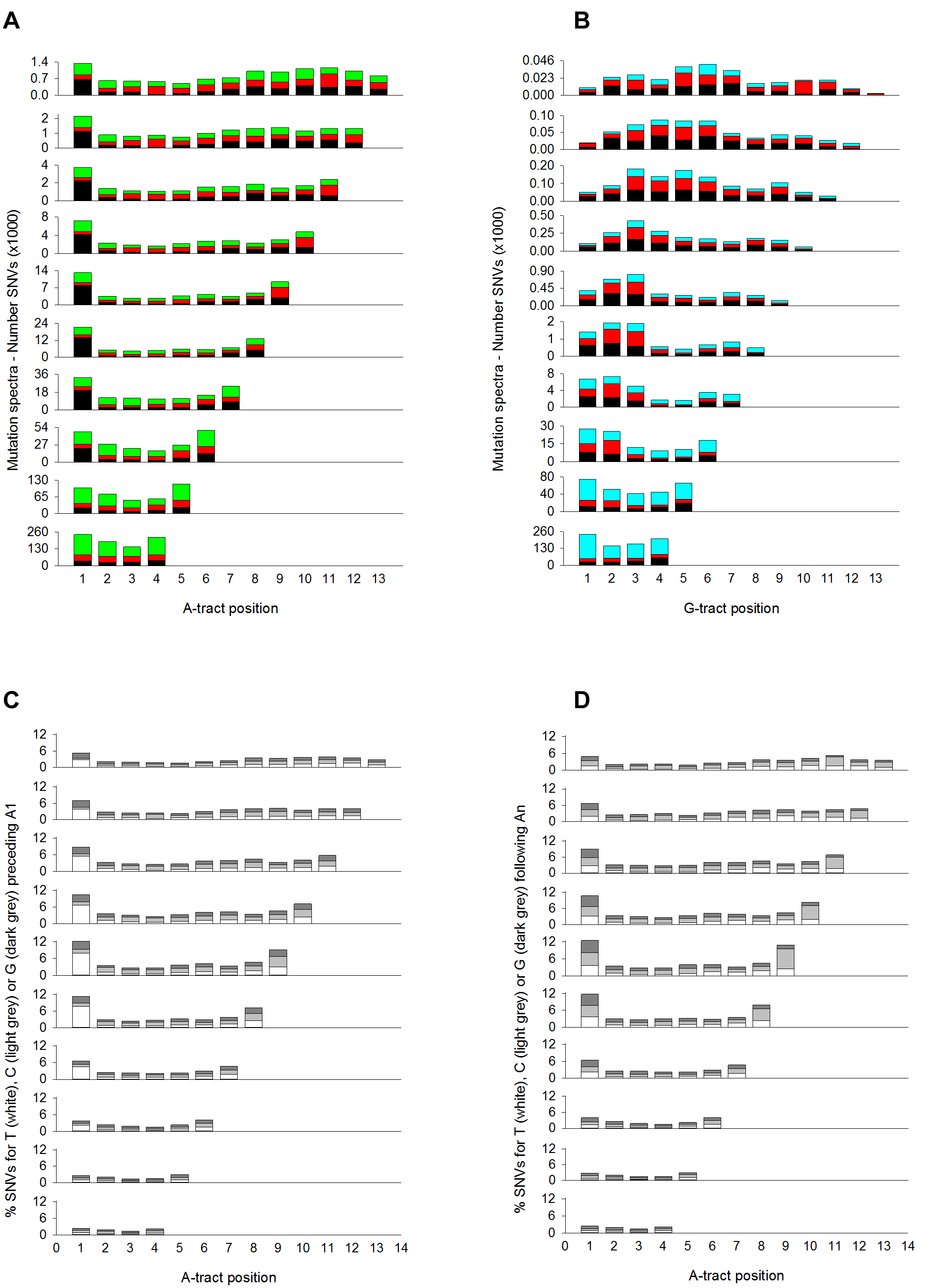


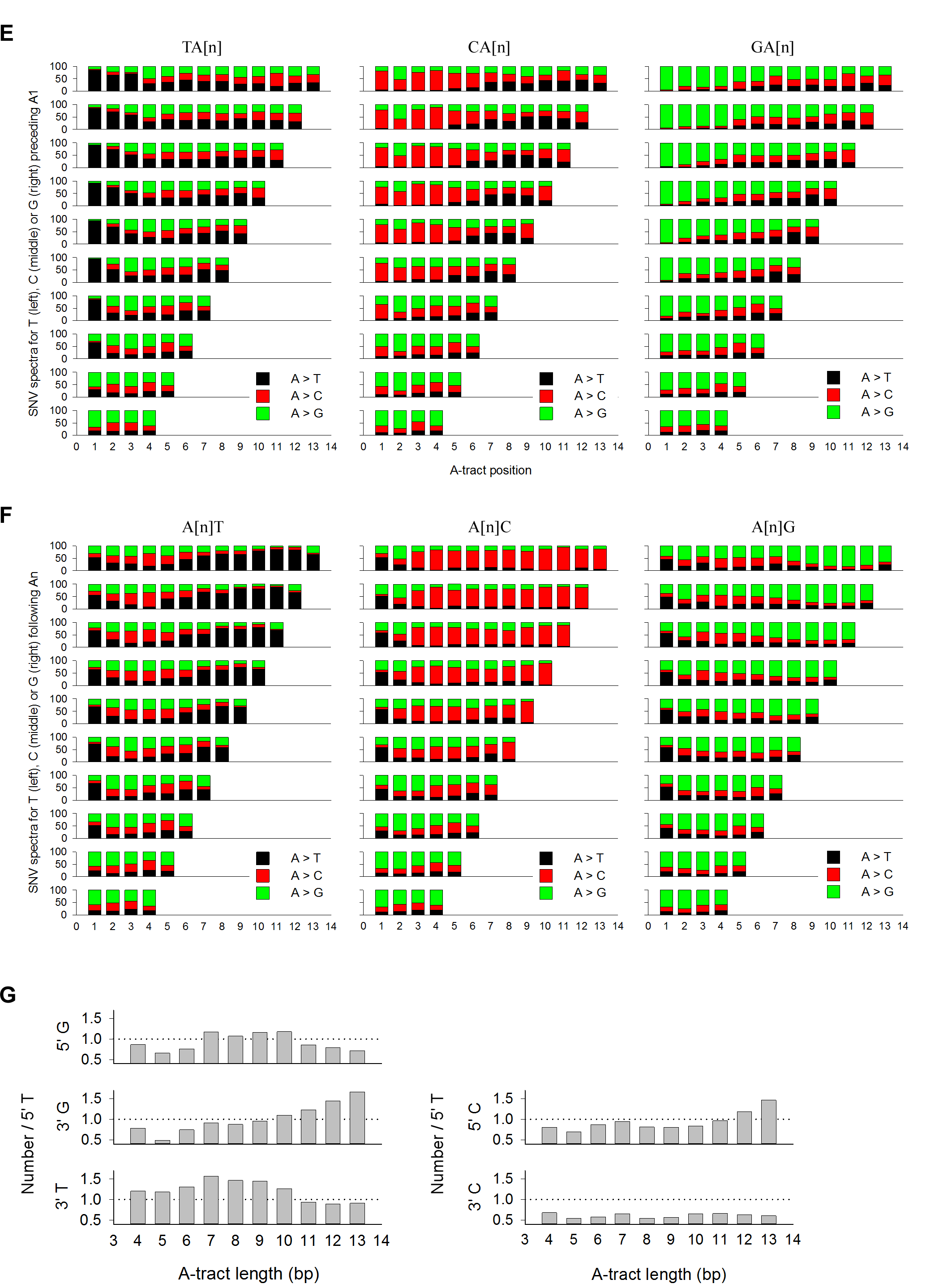


**Supplementary Figure S3**


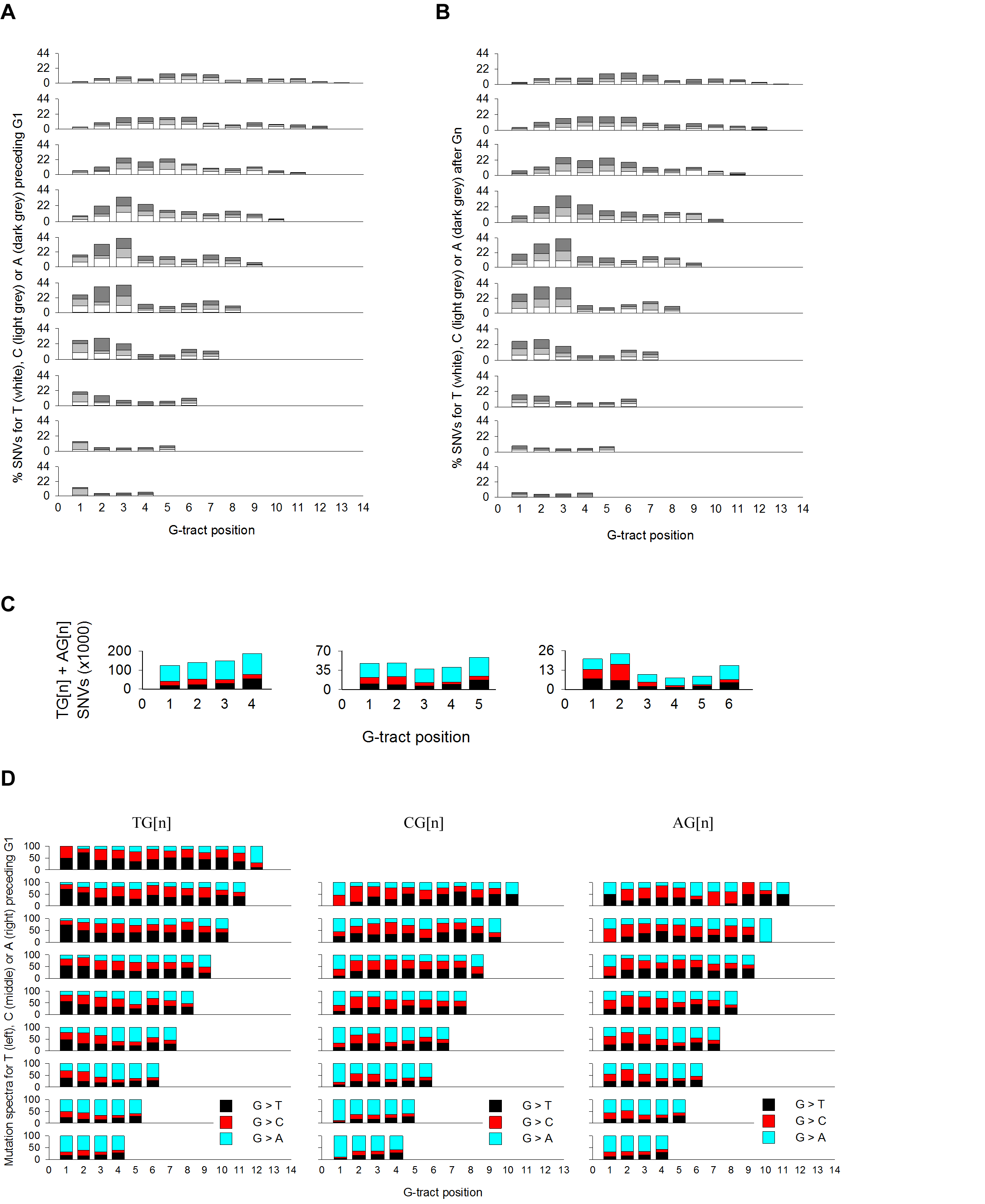


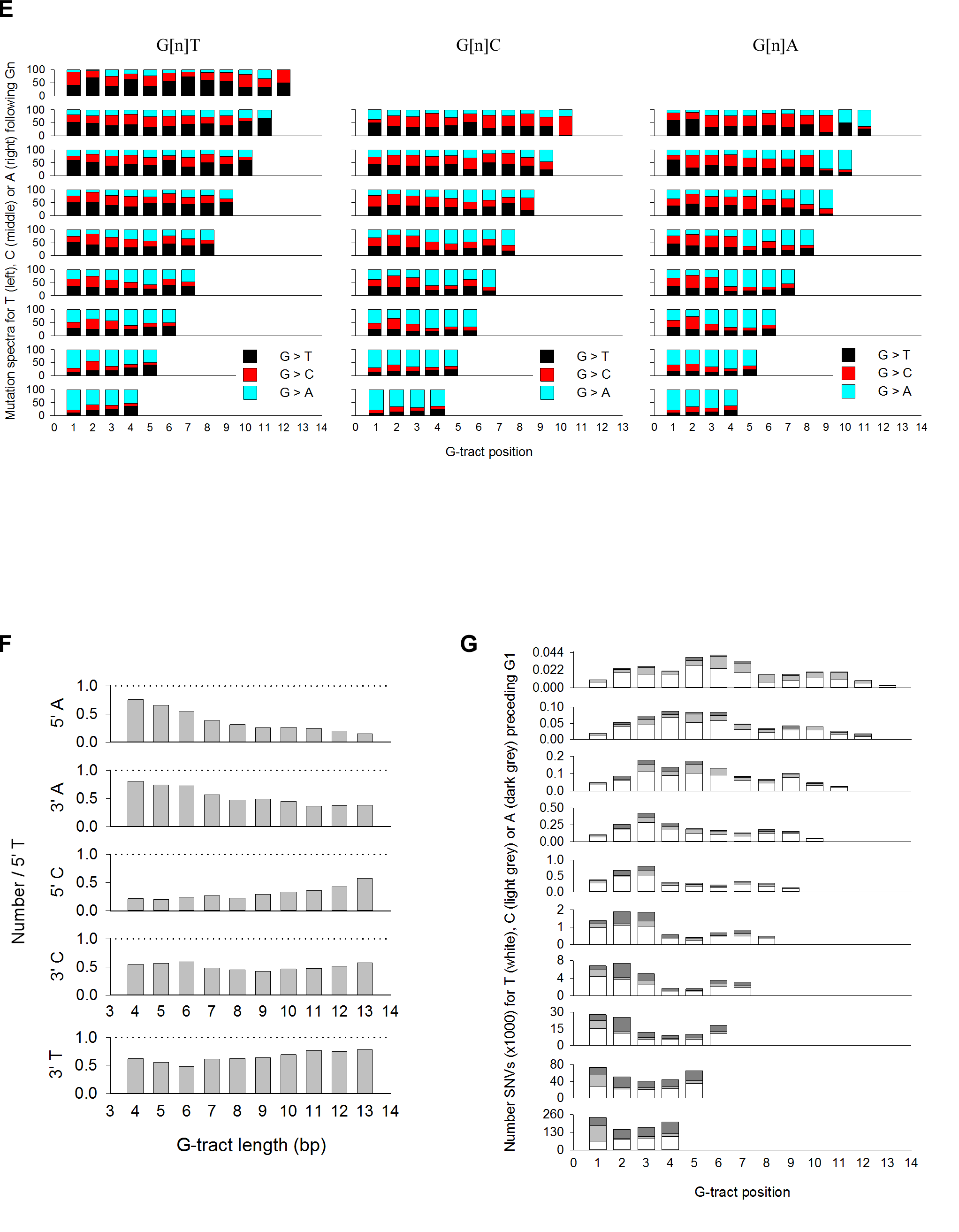


**Supplementary Figure S4**


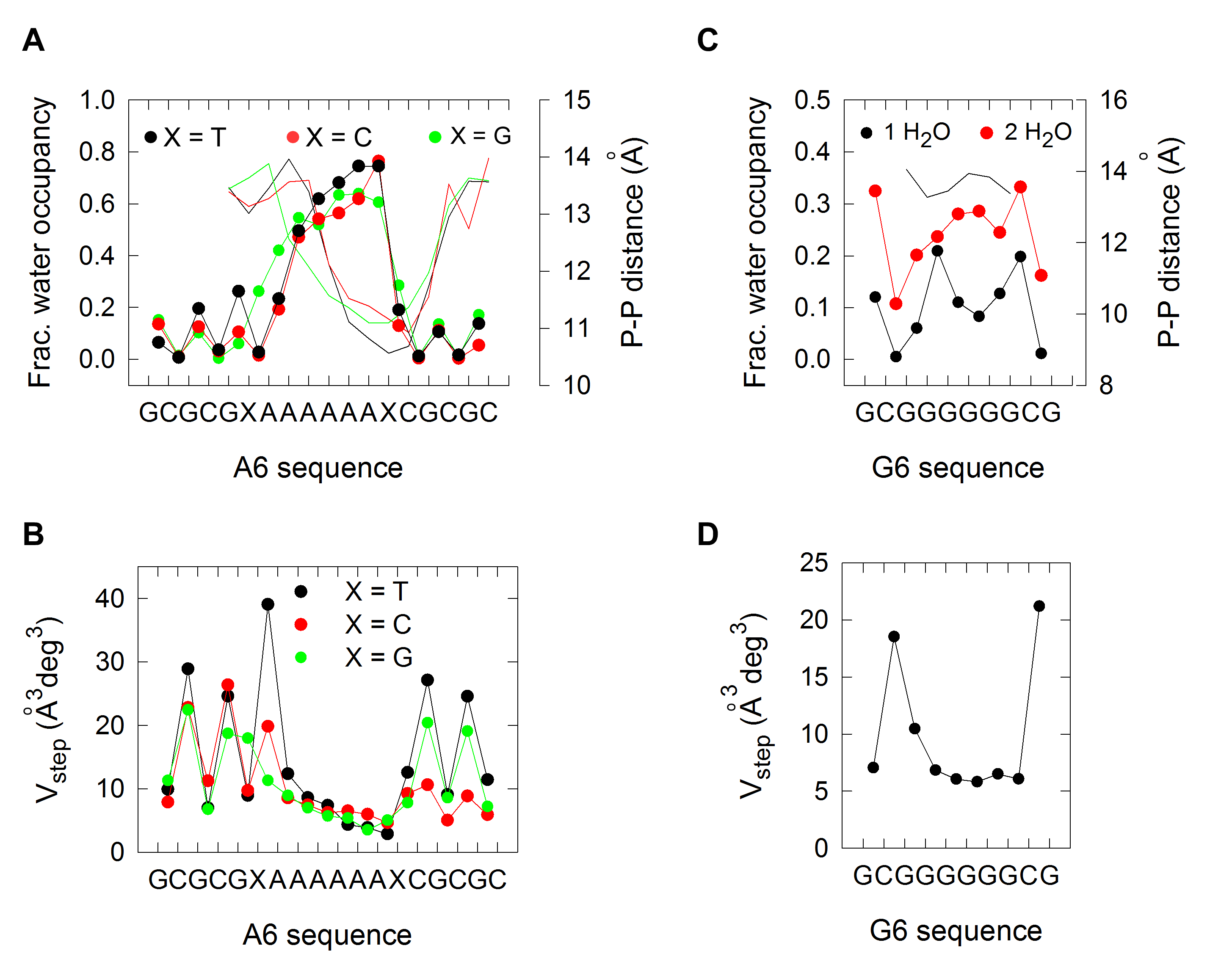


**Supplementary Figure S5**


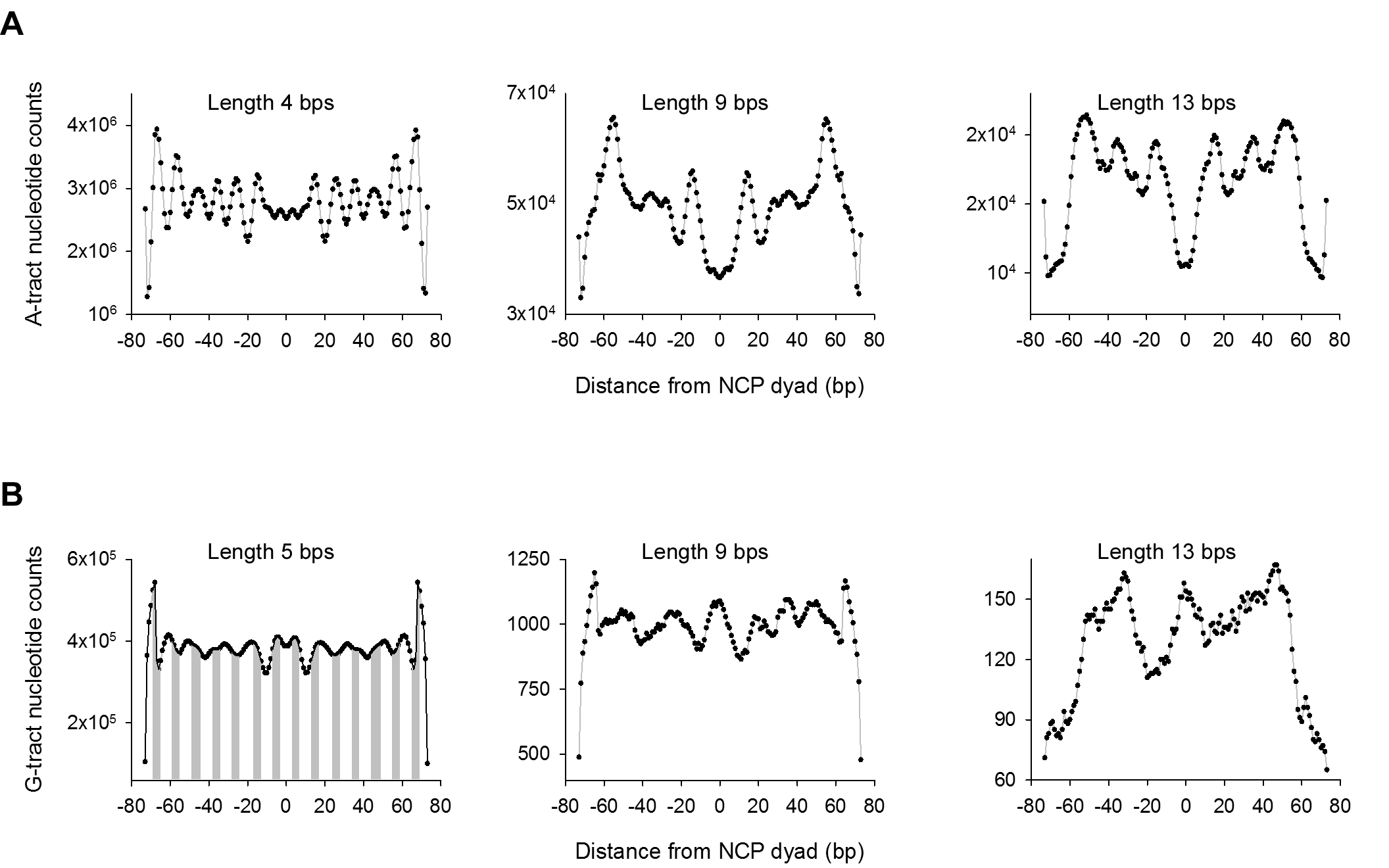


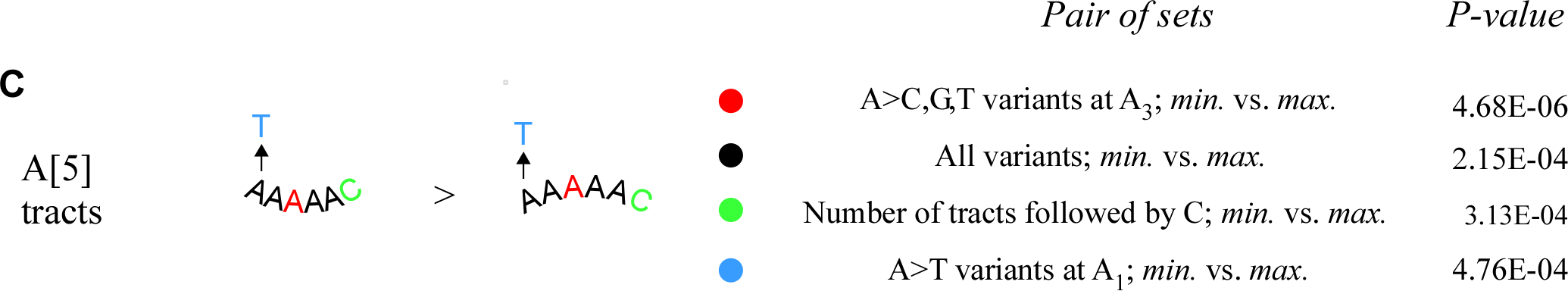


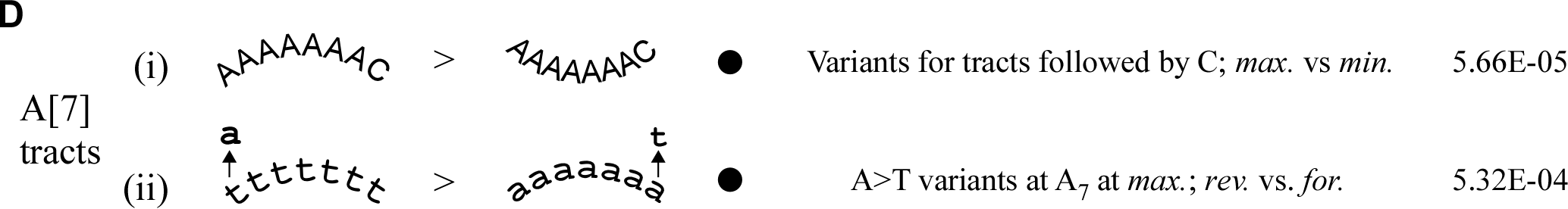


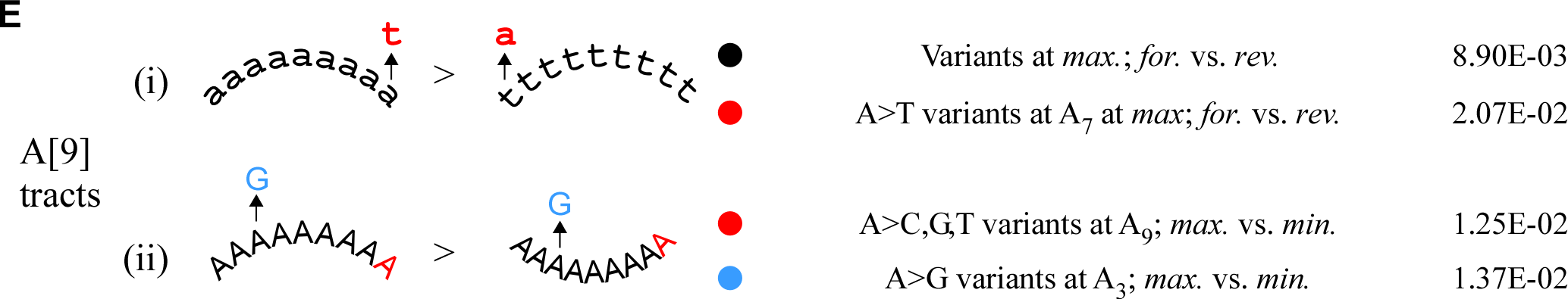


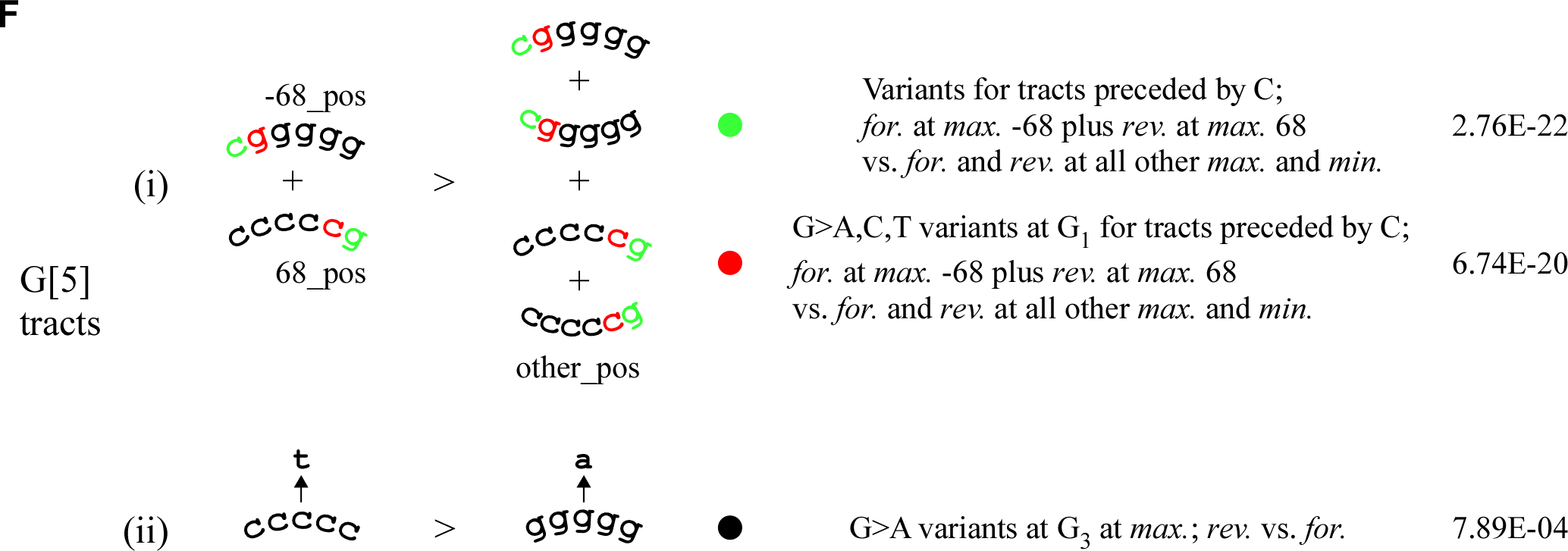


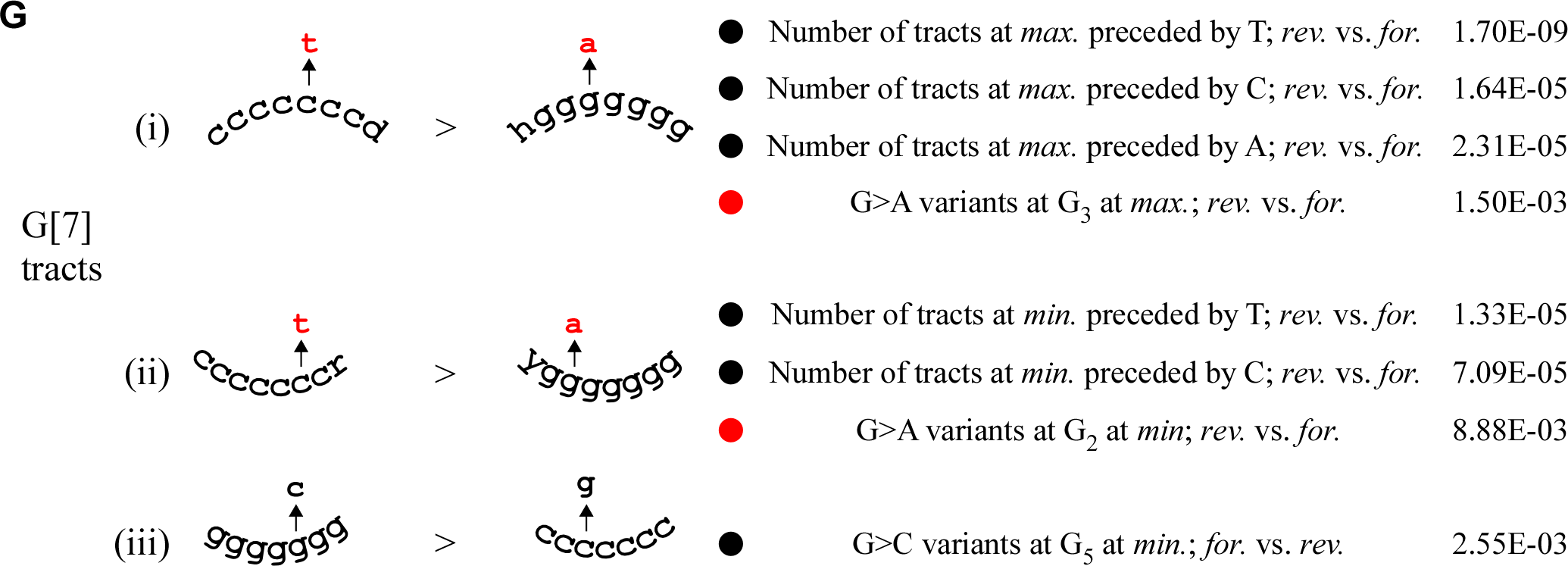


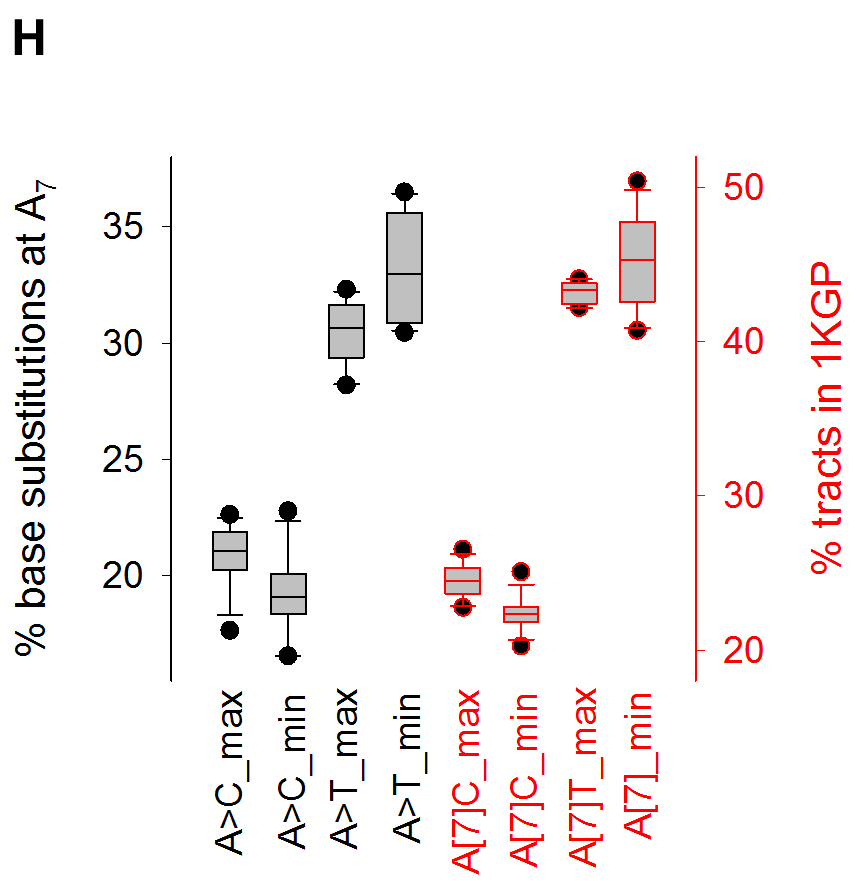


**Supplementary Figure S6**

**
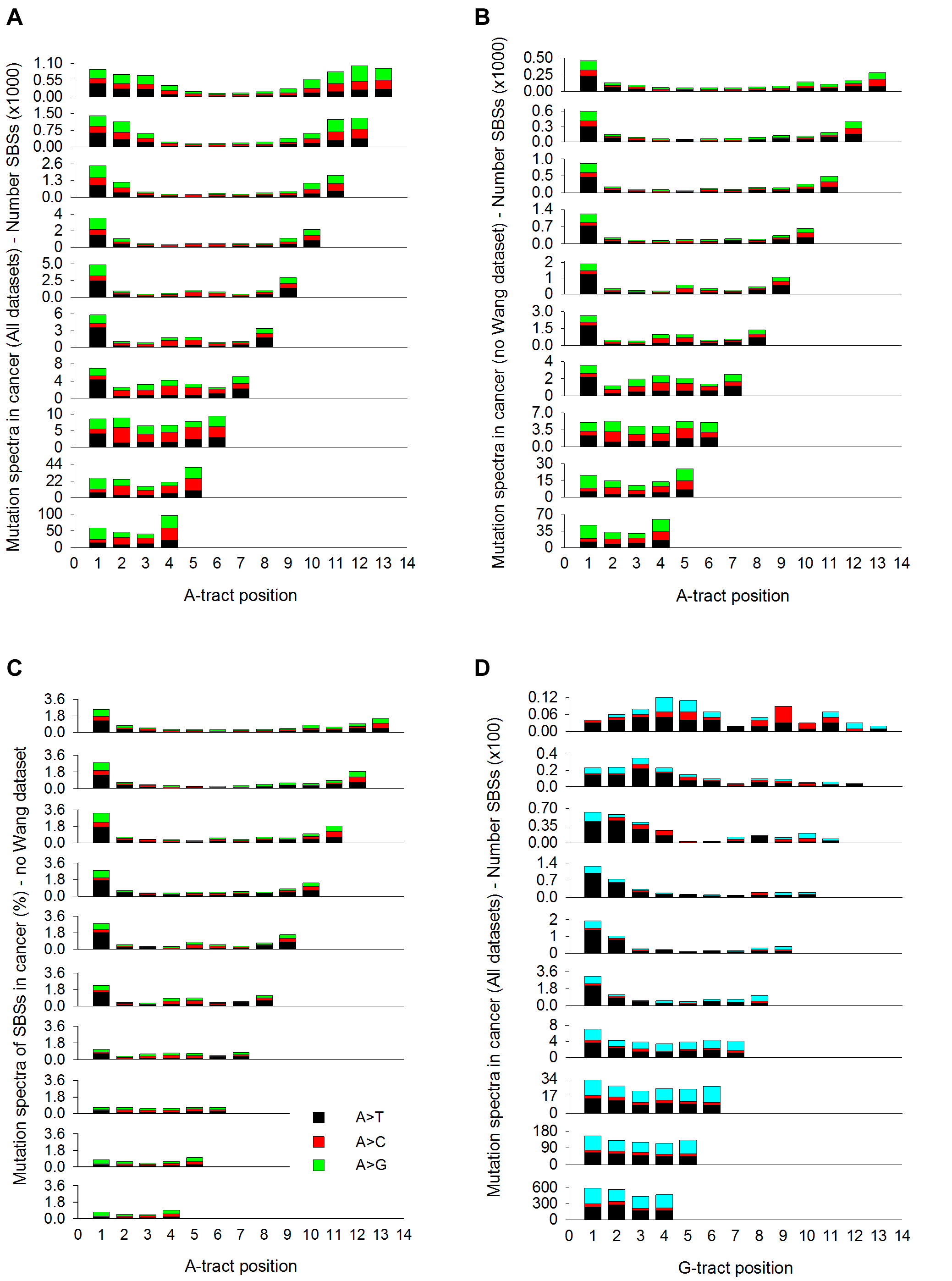
**


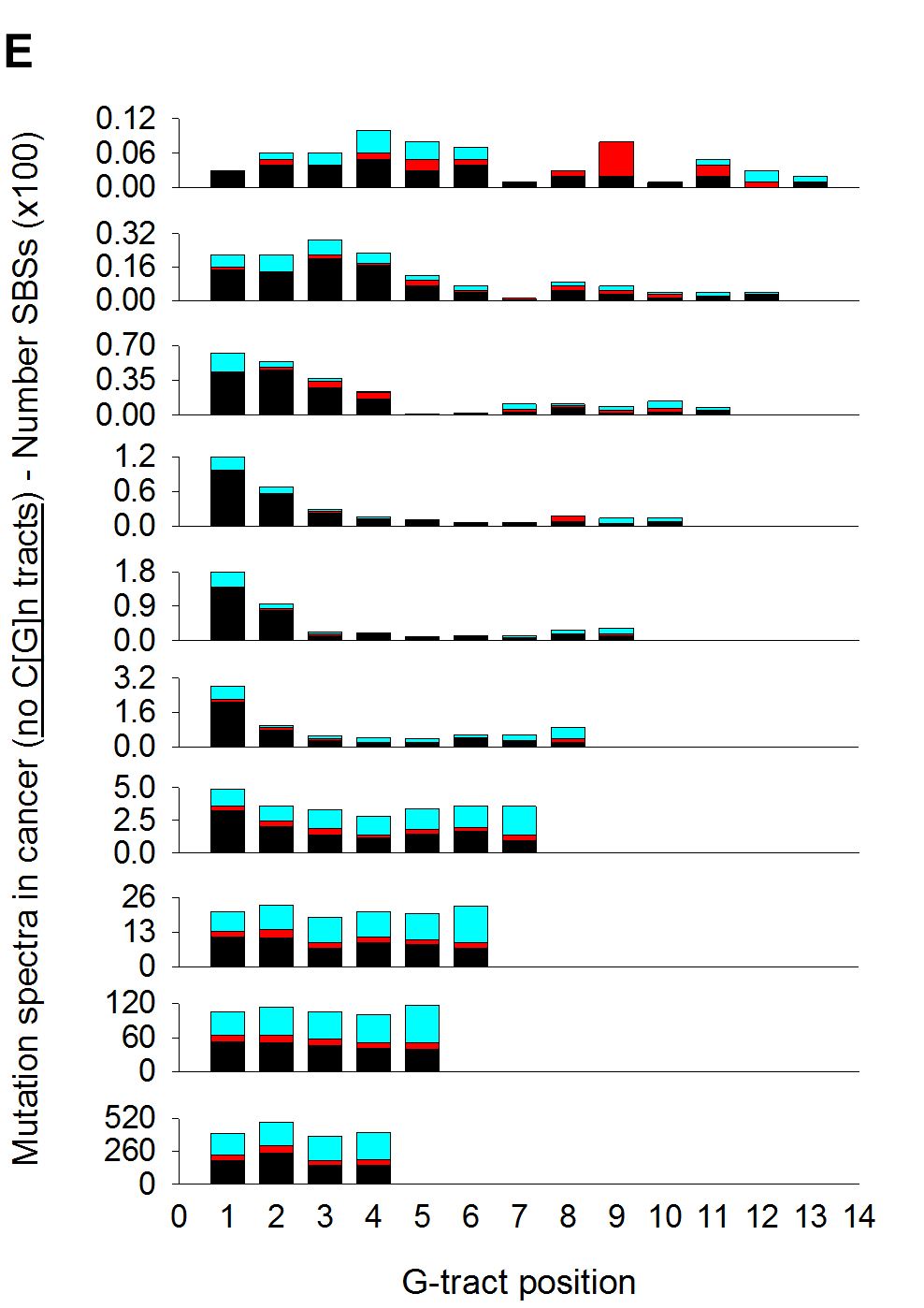


**F**


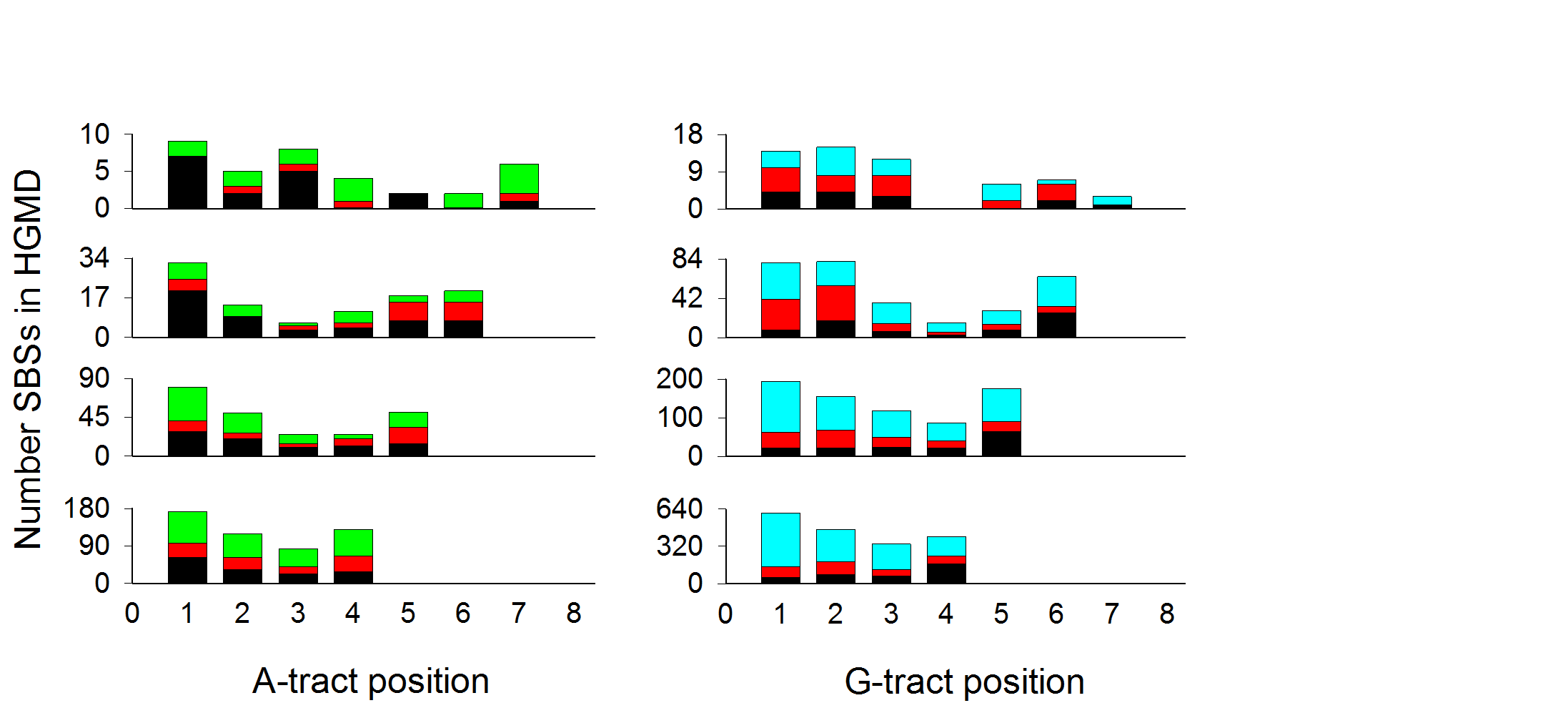


**LEGENDS TO SUPPLEMENTARY FIGURES**

**Supplementary Figure S1. Number of Mononucleotide Tracts and SNVs Is Affected by Flanking Sequence Composition**

A) Logarithmic plot of the number of A-tracts in hg19 flanked 5´ and 3´ by Ts (black), Gs (green) or Cs (red) as a function of tract length. B) Logarithmic plot of the number of G-tracts in hg19 flanked 5´ and 3´ by Ts (black), As (cyan) or Cs (red) as a function of tract length. C) Normalized fractions of SNVs in the 1KGP dataset at A-tracts flanked 5´ and 3´ by Ts (black), Gs (green) or Cs (red) as a function of tract length. D) Normalized fractions of SNVs in the 1KGP dataset at G-tracts flanked 5´ and 3´ by Ts (black), As (cyan) or Cs (red) as a function of tract length. E) Normalized fractions of SNVs at 5´ and 3´ bp flanking A-tracts (closed circles) and G-tracts (open circles) in the 1KGP dataset. Fractions were obtained by dividing the number SNVs by both the number of tracts in hg19 and by 2, to account for the 5´ and 3´ flanks.

**Supplementary Figure S2. Start/End bps Are SNV Hotspots at A-Tracts and Mutation Spectra Follow Flanking Sequence Composition**

A) Mutation spectra for A-tracts. Total number (x1000) of A→T (black), A→C (red) and A→G (green) SNVs in the 1KGP dataset at each position along the tracts. B) Mutation spectra for G-tracts. Total number (x1000) of G→T (black), G→C (red) and G→A (cyan) SNVs in the 1KGP dataset at each position along the tracts. C) Percentage of SNVs in the 1KGP dataset at A-tracts preceded 5´ by a T (white), C (light grey) or G (dark grey) at each position along the tracts. Because normalization was performed by dividing the number of SNVs by the number of T[An], CA[n] or GA[n] tracts, the y-axis scale is ~3-fold greater than in Figure 2A. D) Percentage of SNVs in the 1KGP dataset at A-tracts followed 3´ by a T (white), C (light grey) or G (dark grey) at each position along the tracts. E) Mutation spectra in percentages of A→T (black), A→C (red) and A→G (green) for A-tracts preceded 5´ by a T (TA[n], left), C (CA[n]), middle) and G (GA[n], right) as a function of tract length. F) Mutation spectra in percentages of A→T (black), A→C (red) and A→G (green) for A-tracts followed 3´ by a T (A[n]T, left), C (A[n]C), middle) and G (A[n]G, right) as a function of tract length. G) Number of A-tracts length 4 to 13 bps in hg19 preceded 5´ or followed 3´ by T, C, or G, normalized by dividing for the number of A-tracts preceded 5´ by a T.

**Supplementary Figure S3. SNV Hotspots Occur within G-Tracts and Mutation Spectra Are Insensitive to Flanking Sequence Composition**

A) Percentage of SNVs in the 1KGP dataset at G-tracts preceded 5´ by a T (white), C (light grey) or A (dark grey) at each position along the tracts. B) Percentage of SNVs in the 1KGP dataset at G-tracts followed 3´ by a T (white), C (light grey) or A (dark grey) at each position along the tracts. C) Number of G→T (black), G→C (red) and G→A (cyan) SNVs in the 1KGP dataset for G-tracts length 4 – 6 preceded by A (AG[n]) or T (TG[n], but not C (CG[n]). D) Mutation spectra in percentages of G→T (black), G→C (red) and G→A (cyan) for G-tracts preceded 5´ by a T (TG[n], left), C (CG[n]), middle) and A (AG[n], right) as a function of tract length. E) Mutation spectra in percentages of G→T (black), G→C (red) and G→A (cyan) for G-tracts followed 3´ by a T (G[n]T, left), C (G[n]C), middle) and A (G[n]A, right) as a function of tract length. F) Number of G-tracts length 4 to 13 bps in hg19 preceded 5´ or followed 3´ by T, C, or A, normalized by dividing for the number of G-tracts preceded 5´ by a T. G) Number of SNVs in the 1KGP dataset at G-tracts preceded 5´ by a T (white), C (light grey) or A (dark grey) at each position along the tracts.

**Supplementary Figure S4. Molecular Dynamics Simulation of A[6] and G[6] DNA Fragments**

A) Fraction of one-water bridge occupancy (left axis) for A[6] DNA fragments flanked 5´ and 3´ by a T (black circles), C (red circles) or G (green circles) from MD simulations. Minor groove widths (right axis), as determined from intrastrand phosphate-to-phosphate distances. B) V_step_ for A[6] DNA fragments, determined as describe in the legend to Figure 4B. C) Fraction of one- (black circles) and two-water (red circles) bridge occupancy (left axis) for G[6] DNA fragments from MD simulations. Minor groove widths (right axis) were assessed from intrastrand phosphate-to-phosphate distances. D) V_step_ for G[6] DNA sequences, determined as described in the legend to Figure 4B.

**Supplementary Figure S5. Positioning along Nucleosome Core Particles Influences Mutation Rates at Mononucleotide Tracts**

A) Counts of bps of A-tracts lengths 4, 9 and 13 bps overlapping NCPs genomic regions as a function of distance from the histone octamer dyad axes. B) Counts of bps of G-tracts lengths 5, 9 and 13 bps overlapping NCPs genomic regions as a function of distance from the histone octamer dyad axes. Minor groove-inward-facing regions for G-tracts length 5 (grey shading), identified according to the X-ray crystal structure of NCP147, do not coincide with peak areas. C – G) Representative results for A- and G-tracts along NCPs. C) A-tracts of length 5 bps whose major grooves face towards the NCP (tracts centered at minima) undergo greater percentages of SNVs than those whose minor grooves face towards the NCPs (tracts centered at maxima) (black), with the greatest increases in SNVs for A→B at the center position, A_3_, (red) and for A→T at the first position, A_1_ (blue). For tracts followed by a C in hg19, a greater proportion is positioned with the major, rather than the minor, groove facing towards the NCPs (green). D) (i) A-tracts of length 7 bps whose minor grooves face towards the NCP (tracts centered at maxima) undergo greater percentages of SNVs than those whose major grooves face towards the NCPs (tracts centered at minima) (black). (ii) For tracts with minor grooves facing inwards, those in the reverse orientation (T-rich strand in Figure 1A) undergo a greater percentage of A→T SNVs at the last position, A_7_, than those in the forward orientation (A-rich strand in Figure 1A) (black). E) (i) For A-tracts of length 9 bps with minor grooves facing inwards, those in the forward orientation display greater percentages of SNVs than those in the reverse orientation (black), particularly at the last, A_9_, position (red). (ii) A-tracts whose minor grooves face inwards display greater percentages of A→T (blue) at A_3_ and A→B at A_9_ (red) than those whose major grooves face inwards. F) (i) For G-tracts of length 5 bps and preceded by a C, the percentages of SNVs (green) and G→A at G_1_ (red) for tracts in the forward orientation and centered at maxima -68 from the NCP dyad axis together with those in the reverse orientation and centered at maxima 68 (i.e. the most external NCP positions) are greater than for all other tracts. A similar result is revealed for G-tracts of length 7 bps between tracts centered at the most external NCP positions (-66 and 66) and all other NCP positions. (ii) For G-tracts of length 5 bps and centered at maxima, those in the reverse orientation display greater G→A SNVs at G_3_ than those in the forward orientation (black). G) (i) In hg19, the percentage of G-tracts of length 7 bps preceded by T, C, or A (black) centered at maxima along the NCP surface is greater for tracts in the reverse orientation than for those in the forward orientation; likewise, in the 1KGP dataset, tracts at maxima in the reverse orientation display greater percentages of G→A SNVs at G_3_ than for those in the forward orientation (red). (ii) In hg19, the percentage of tracts preceded by T or C (black) centered at minima along the NCP surface is greater for tracts in the reverse orientation than for those in the forward orientation; in the 1KGP dataset, tracts at minima in the reverse orientation display greater percentages of G→A SNVs at G_2_ than for those in the forward orientation (red). (iii) Tracts at minima in the forward orientation display greater percentages of G→C SNVs at G_5_ than for those in the reverse orientation (black). H) Whisker plots of percent base substitutions (left axis) at A_7_ for A-tracts length 7 bps centered at maxima and minima (black) along NCPs. Percent (right axis) of the number of A-tracts length 7 bps followed by C or T in hg19 centered at maxima and minima (red).

**Supplementary Figure S6. Mononucleotide Mutation Spectra in Cancer Genomes and Human Inherited Disease**

A) Mutation spectra at A-tracts in cancer. Total number (x1000) of A→T (black), A→C (red) and A→G (green) SBSs at each position along the tracts in all cancer datasets. B) Mutation spectra at A-tracts in selected cancer datasets. Total number (x1000) of A→T (black), A→C (red) and A→G (green) SBSs at each position along the tracts in the cancer datasets, except the gastric cancer dataset from Wang et al., 2014. C) Mutation spectra for SBSs at A-tracts in selected cancer datasets. Percent values were obtained by dividing the number of SBSs at each position by the number of tracts in hg19 and then multiplying by 4.6731, i.e. the ratio of the percent for the tracts length 4 in the 1KGP to that of the tracts also length 4 in the cancer datasets. A→T (black), A→C (red) and A→G (green). D) Mutation spectra at G-tracts in cancer. Total number (x100) of G→T (black), G→C (red) and G→A (cyan) SBSs at each position along the tracts in all cancer datasets. E) Mutation spectra for A[G]n and T[G]n tracts in cancer. Total number (x100) of G→T (black), G→C (red) and G→A (cyan) SBSs at each position along the tracts in all cancer datasets. F) Mutation spectra in HGMD. Total number of A→T (black), A→C (red) and A→G (green) SBSs at each position along A-tracts (left). Total number of G→T (black), G→C (red) and G→A (cyan) SBSs at each position along G-tracts (right).
